# Effects of day length- and temperature-regulated genes on annual transcriptome dynamics in Japanese cedar (*Cryptomeria japonica* D. Don), a gymnosperm indeterminate species

**DOI:** 10.1371/journal.pone.0229843

**Published:** 2020-03-09

**Authors:** Mine Nose, Manabu Kurita, Miho Tamura, Michinari Matsushita, Yuichiro Hiraoka, Taiichi Iki, So Hanaoka, Kentaro Mishima, Miyoko Tsubomura, Atsushi Watanabe

**Affiliations:** 1 Forest Tree Breeding Center, Forestry and Forest Products Research Institute, Forest Research and Management Organization, Hitachi, Ibaraki, Japan; 2 Department of Forest Environmental Sciences, Faculty of Agriculture, Kyushu University, Fukuoka, Japan; University of North Carolina at Greensboro, UNITED STATES

## Abstract

Seasonal phenomena in plants are primarily affected by day length and temperature. The shoot transcriptomes of trees grown in the field and a controlled-environment chamber were compared to characterize genes that control annual rhythms and the effects of day length- and temperature-regulated genes in the gymnosperm Japanese cedar (*Cryptomeria japonica* D. Don), which exhibits seasonally indeterminate growth. Annual transcriptome dynamics were clearly demonstrated by principal component analysis using microarray data obtained under field-grown conditions. Analysis of microarray data from trees grown in a controlled chamber identified 2,314 targets exhibiting significantly different expression patterns under short-day (SD) and long-day conditions, and 2,045 targets exhibited significantly different expression patterns at 15°C (LT; low temperature) versus 25°C. Interestingly, although growth was suppressed under both SD and LT conditions, approximately 80% of the SD- and LT-regulated targets differed, suggesting that each factor plays a unique role in the annual cycle. The top 1,000 up-regulated targets in the growth/dormant period in the field coincided with more than 50% of the SD- and LT-regulated targets, and gene co-expression network analysis of the annual transcriptome indicated a close relationship between the SD- and LT-regulated targets. These results indicate that the respective effects of day length and temperature interact to control annual transcriptome dynamics. Well-known upstream genes of signaling pathways responsive to environmental conditions, such as the core clock (*LHY/CjLHYb* and *CCA1/CjLHYa*) and PEBP family (*MFT*) genes, exhibited unique expression patterns in Japanese cedar compared with previous reports in other species, suggesting that these genes control differences in seasonal regulation mechanisms between species. The results of this study provide new insights into seasonal regulation of transcription in Japanese cedar.

## Background

Day length and temperature, which exhibit particularly large annual changes, have a marked influence on seasonal phenomena, such as bud flush and formation, growth pattern, and dormancy induction [1–6]. A number of previous reports have described how annual cycles are controlled at the transcript level and the effects of day length and temperature in the model tree, *Populus* [5–9]. Although the molecular mechanisms regulating seasonal cycles in angiosperm trees such as *Populus* have been extensively studied, these cycles may be controlled by different mechanisms in gymnosperm trees, given that angiosperms and gymnosperms separated evolutionarily 300 million years ago [10]. Indeed, recent studies of gymnosperm trees described unique characteristics of a number of genes that function as important regulators of environmental responses. For example, while the expression of homologs of *FT* (*FLOWERING LOCUS T*), which regulates flowering time and seasonal growth, decreased in *Populus* (*PtFT1*), it increased in *Picea abies* (Norway spruce, *PaFT4*) during the period of growth cessation [11–14]. The expression of core clock genes, which play roles in adaptation to day length and temperature changes and the regulation of seasonal phenomena [15, 16], was shown to be arrested under conditions of continuous light or dark in gymnosperm conifers, in contrast to angiosperms [17, 18]. Differences in diurnal rhythms of clock genes between *Arabidopsis* and *Pseudotsuga menziesii* (Douglas-fir) have also been reported [19]. Collectively, these data suggest the importance of studying gymnosperms to understand their unique seasonal regulatory mechanisms.

To date, few studies of evergreen coniferous species of gymnosperms have used time-series transcriptome analyses to investigate transcripts of needles, the key perennial organ that senses changes in environmental conditions, in order to elucidate seasonal changes and the effects of environmental factors [19–21]. Global changes in gene expression from late summer to early winter (August to December) have been reported in *Picea sitchensis* (Sitka spruce) using microarrays [20]. In *P*. *menziesii*, >80% of all identified transcripts were reportedly responsive to variations in environmental conditions during the summer (May to September) in the field [21], and analysis of annual samples indicated that 58.7% of transcripts exhibited a circannual cycle [19]. Although these studies reported seasonal dynamics and estimated the effect of environmental factors on transcript levels using data collected under field conditions [19, 21], there are no published reports of studies verifying the effects of individual environmental factors on seasonal transcriptome dynamics. As multiple environmental factors, including day length and temperature, change synchronously under natural conditions, it is difficult to estimate the effect of a single factor. Comparative studies of trees grown in the field and controlled-environment chambers could enable clearer estimations of the roles of individual environmental factors in regulating annual transcriptome dynamics.

Japanese cedar (*Cryptomeria japonica* D. Don), a major forestry species in Japan, exhibits various unique characteristics compared with other coniferous species. Japanese cedar grows continuously until environmental or internal factors cause growth to cease (indeterminate species), whereas the amount of annual growth is regulated endogenously and environmental factors have a minor effect on the timing of growth cessation in many other coniferous species (determinant species), such as those of the genera *Picea* and *Pinus* [4, 16, 22]. Different mechanisms may regulate the growth of indeterminate and determinate species in response to environmental factors and control annual growth, particularly during the transition to dormancy. A previous report on Japanese cedar indicated that annual growth is influenced primarily by photoperiod and temperature [3]. The duration of growth (or conversely, the timing of dormancy induction) is important during preparation for harsh winter conditions. Investigating the contribution of photoperiod and temperature to annual transcriptome dynamics may help elucidate the mechanism by which seasonal phenomena affect the growth and environmental adaptation of indeterminate coniferous species such as Japanese cedar.

The objective of this study was to characterize the features of annual rhythms and to identify the respective effects of day length and temperature on annual rhythms in Japanese cedar by analyzing the transcriptome. The genes that exhibited annual expression rhythms under field conditions were presumed to contain the genes influenced by the respective effects of day length and temperature. We had hypothesized that a gymnosperm indeterminate species may have unique regulatory mechanisms compared with other angiosperms and/or determinate tree species reported previously. Therefore, we focused particularly on features at the transition from growth to dormancy, such as growth suppression and preparation for winter, and the regulatory mechanisms of clock genes.

First, annual transcriptome dynamics of field-grown trees were investigated by analyzing shoot samples collected throughout the year. Second, as it can be difficult to determine the effects of individual environmental factors under natural conditions, cuttings grown in a controlled-environment chamber under different day lengths and temperatures were examined using time series microarray analysis. The respective effects of day length and temperature and regulated targets were identified. Finally, the contribution of day length- and temperature-regulated genes to annual transcriptome dynamics were elucidated by comparing the results obtained under field and experimental conditions. The relationship of targets regulated by the respective effects of day length and temperature in annual transcriptome dynamics was predicted via co-expression gene network analysis.

## Methods

### Plant materials and samples

#### Measuring annual height increase in Japanese cedar

The annual growth of Japanese cedar was measured to estimate the relationship between growth and environmental conditions. The temperature of the controlled-environment chamber was determined based on these data. Because of tree size, 2-year-old trees were used for accurate measurements. The height of six cuttings of a Japanese cedar plus tree clone (Godai-1) planted in 2013 at the Forest Tree Breeding Center (FTBC), Forestry and Forest Products Research Institute (FFPRI, Hitachi, Ibaraki, Japan), was measured 19 times between February 18, 2014, and December 10, 2014. Growth rate was calculated by dividing growth at each time point by the initial value obtained on February 18, 2014.

#### Annual time series samples for transcriptome analysis

To analyze seasonal transcriptome dynamics under natural conditions, annual time series samples were collected at 8:00 am on 12 dates over a period of 1 year (from June 2013 to June 2014) from eight cuttings of a clone Godai-1 planted in 1999 at the FTBC (Table 1). Annual changes in day length and temperature at Hitachi are shown in S1 Fig. A 10-cm portion of the lateral branch apex was collected from three individual trees in random order at each time point (36 samples total).

**Table 1:** Description of samples used in this study.

name of samples	conditions	day of sampling		time points	replicates	number of samples
annual time series samples	natural conditions* (Hitachi, Ibaraki, Japan)	24-Jun-13	5-Aug-13	12	3	36
		30-Sep-13	30-Oct-13			
		26-Nov-13	24-Dec-13			
		30-Jan-14	21-Feb-14			
		11-Mar-14	9-Apr-14			
		9-May-14	18-Jun-14			
experimental time series samples						
day length						
short day (SD)	8 h of light and 16 h of darkness, 25°C	7, 21, 35, 49 and 70 days		5	2	10
long day (LD)	16 h of light and 8 h of darkness, 25°C	0, 7, 21, 35, 49 and 70 days		6	2	12
temperature						
low temperature (LT)	16 h of light and 8 h of darkness, 15°C	7, 21, 42, 63 and 90 days		5	2	10
high temperature (HT)	16 h of light and 8 h of darkness, 25°C	0, 7, 21, 42, 63 and 90 days		6	2	12

*Annual changes in day length and temperature at Hitachi are shown in S1 Fig.

#### Experimental time series samples for transcriptome analysis

One-year-old cuttings of the Godai-1 clone were potted on March 4, 2014, and grown in a greenhouse at the FTBC. To evaluate the effect of day length, 16 cuttings were repotted on July 7, 2014, and transferred to chambers and cultivated under long-day (LD) conditions (16 h of light and 8 h of darkness) at 25°C (Table 1). On August 12, 2014, nine pots were transferred to chambers and cultivated under short-day (SD) conditions (8 h of light and 16 h of darkness) at 25°C, and seven pots were left under the LD conditions. A 10-cm portion of the lateral branch apex was harvested from two cuttings at 0, 7, 21, 35, 49, and 70 days.

To analyze the effect of temperature, the 18 cuttings were transferred from the greenhouse and placed under high-temperature (HT; 25°C under 16 h of light and 8 h of darkness) conditions on August 5, 2014, and repotted on October 24, 2014. On November 28, 2014, nine pots were transferred to low-temperature (LT; 15°C under 16 h of light and 8 h of darkness) conditions, and nine pots were left at HT (Table 1). Based on the annual growth pattern (S2 Fig), we selected 25°C as the control temperature, as this is the average temperature in July, when Japanese cedar exhibits continuous growth, and 15°C was selected as the low temperature, as this is the average temperature in November, when Japanese cedar have stopped growing. In addition, 15°C is the temperature at which Japanese cedar starts to acquire cold hardiness [23]. A 10-cm portion of the lateral branch apex was harvested from two cuttings grown at HT and LT at 0, 7, 21, 42, 63, and 90 days after transfer.

The height of three individual cuttings was measured weekly. Lateral crown images of the three cuttings were captured using a WG-II digital camera (Pentax, Tokyo, Japan), and the projected area of the images was analyzed using ImageJ 64 software (http://rsbweb.nih.gov/ij/) to estimate the shoot growth.

### RNA extraction and microarray gene expression analysis

All samples were immediately frozen in liquid nitrogen and stored at −80°C until use. Total RNA was extracted using an RNeasy Plant Mini Kit (Qiagen, Hilden, Germany) according to a previous report [24] from 500 mg of seasonal samples collected from trees planted in the field. DNase digestion was performed on-column using an RNase-free DNase set (Qiagen). Total RNA from cuttings placed in the controlled-environment chamber was first extracted by LiCl precipitation [25], and then extracted again using an RNeasy Plant Mini Kit as described above, since purified RNA was difficult to isolate using only an RNeasy Plant Mini Kit. A NanoDrop 1000 spectrophotometer (Thermo Scientific, Waltham, MA, USA) was used to measure the RNA concentration. RNA integrity was assessed using an Agilent 2100 Bioanalyzer (Agilent Technologies, Mississauga, ON, Canada).

Microarray probes were designed based on isotig sequences from next-generation sequencing (NGS) data collected from analyses of various organs (wood cambium, treetop, shoots, and male strobili) of Japanese cedar at several developmental stages and in different seasons using a Roche GS-FLX system, as reported previously [26–28]. The NGS data (total 552.4 Mbp, approximately 1.3 million reads) were assembled into 22,250 isotigs as described in a previous report [28]. SurePrint G3 Gene Expression Custom 8×60K Array probes (Agilent Technologies) consisted of three probe sets corresponding to 22,194 sequences designed using the base composition methodology of the eArray tool (Agilent Technologies) using the default settings. Gene annotations represented the top-scoring BLASTX hits determined using each sequence’s predicted protein product as a query against the *Arabidopsis* protein database TAIR10-pep-20101214 in The Arabidopsis Information Resource (http://www.arabidopsis.org) with a threshold e-value of e-5 using CLC Genomic Workbench software package, version 4.1.1 (CLC bio, Aarhus, Denmark).

Total RNA (200 ng) was used for microarray analyses. cRNA was amplified and labeled using a Low Input Quick-Amp Labeling Kit (Agilent Technologies), with hybridization and washing performed according to the manufacturer’s instructions. The slides were scanned on a SureScan Microarray Scanner G4900DA (Agilent Technologies), and data from the scans were compiled using Agilent Feature Extraction software, version 11.5.1.1 (Agilent Technologies).

### Microarray data analysis

Raw data were normalized using a 75th-percentile shift, and median log_2_-transformed ratios for each time point were normalized to baseline intensity values (normalized intensity values) using GeneSpring software, version 14.5 (Agilent Technologies). A total of 10,439 targets with a raw signal intensity ≥1,000 in 100% of samples for at least one of the 34 conditions were selected for further analysis.

To obtain a high-level overview of gene expression annual dynamics, principal component analysis (PCA) was performed using the 10,439 targets of the 12 seasonal time series data sets (average normalized intensity value of three replicates) using the *prcomp* function in R software (R Development Core Team 2015). Based on the composite scores obtained in the first step, the expression of genes under the experimental conditions (test data) was then compared to expression under natural conditions (training data) using the R function *predict* to estimate the effect of seasonal conditions. To estimate the physiologic difference between growth and dormant periods with respect to the expressed genes, the functional categorizations of the top 1,000 targets with positive and negative component scores in principal component 1 (PC1) were compared to the 10,439 reference targets using the PANTHER Overrepresentation Test (Fisher’s exact test, false-discovery rate [FDR] <0.05) of the PANTHER Classification System (released 20171205, http://pantherdb.org). The unique set of *Arabidopsis* gene IDs (e-value < e-5) was annotated to ‘GO biological process complete’.

To identify genes regulated by day length, the R package maSigPro [29] was used. Significant gene expression profile differences between experimental groups in the time course transcriptome data were identified using maSigPro, based on a regression approach. Targets exhibiting significantly different expression profiles in a time-course experiment (7, 21, 35, 49, and 70 days) under SD and LD conditions were identified using normalized intensity values (quadratic regression, FDR ≤10^−4^, p-value ≤10^−4^, *R*^*2*^ ≥0.7). The differentially expressed targets were classified into four clusters using the hclust function of maSigPro, and median profiles of resulting clusters were plotted using maSigPro to visualize their expression patterns. The same analysis was performed to identify LT-regulated targets using time series data collected under LT and HT conditions (7, 21, 42, 63, and 90 days). The resulting clusters were categorized based on function and compared to the 10,439 reference targets using the PANTHER Overrepresentation Test, as described above.

To demonstrate the relationship between genes that exhibited dramatic expression changes over the course of a year, a co-expression gene network was estimated from 2,000 targets exhibiting the highest standard deviation in normalized intensity value among 36 annual time series samples. Correlation coefficients among the 2,000 targets were calculated using the R function (cor()), and gene pairs exhibiting a high correlation coefficient (cc) were selected (|cc| ≥0.8). The co-expression gene network was illustrated using the R package igraph [30] and laid out using ‘layout_with_mds’ to demonstrate the roles of SD- and LT-regulated genes in the network.

### Quantitative RT-PCR

To assess the reliability of the microarray data by comparing the expression patterns determined using both microarray and quantitative RT-PCR (qRT-PCR) techniques, qRT-PCR analyses were carried out for five genes exhibiting differential expression patterns under natural conditions (*MFT*; *mother of FT and TFL1*, *TEM1*; *tempranillo 1*, *RNA-binding (RRM/RBD/RNP motifs) family protein*, *LHY/CjLHYb*, *HEAT SHOCK PROTEIN 81–1*). The primer pairs were designed using the Oligo software package, version 7 (National Biosciences Inc., Cascade, CO, USA) (S1 Table). First-strand cDNA synthesis and qRT-PCR were performed as described in a previous report [27]. Reaction efficiency was assessed using standard curves based on a 4-fold dilution series of cDNAs synthesized from 1,000 ng of total RNA (1:6 to 1:1,536 dilutions). Each sample was tested independently and in triplicate using all primers. Transcript abundance was normalized to eukaryotic translation initiation factor 4E1 (*EIF4E1*) and protein phosphatase 2A subunit A2 (*PP2AA2*), both of which exhibited expression variance <1.5-fold under all 34 conditions in the microarray data, determined using a method described elsewhere [31]. Data obtained for each time point were compared with data obtained for the sample in the seasonal series collected on June 24, 2013. Very similar expression patterns were obtained for transcripts analyzed using both qRT-PCR and microarray techniques (S3 Fig), suggesting that the data obtained in this study were reliable.

### Phylogenetic and diurnal analysis of expression of the *MFT* gene

Further analyses were performed to examine expression of the *MFT* gene, a member of the phosphatidylethanolamine-binding protein (PEBP) gene family [14], which exhibited an interesting expression pattern in the microarray data. The amino acid sequence of MFT in Japanese cedar was estimated from the nucleic acid sequence of the *NGS* isotig (reCj16177ex_ne:-LSW_isotig16144, e-value 5.1E-84) using CLC Genomics Workbench software, version 11.0 (CLC bio). Phylogenetic analyses were performed using the amino acid sequences of genes homologous to *MFT* from plant species registered in the National Center for Biotechnology Information (NCBI) using ClustalW software, version 2.1, on the DNA Data Bank of Japan (DDBJ) website in default mode (https://clustalw.ddbj.nig.ac.jp/) according to the neighbor-joining method [32]. The phylogenetic tree was illustrated using MEGA X [33].

As *MFT* is known to exhibit circadian rhythms in other species, we performed qRT-PCR analyses to elucidate the diurnal expression pattern of *MFT* in Japanese cedar. Samples were collected every 4 h beginning at 4:00 pm over a period of 2 days in summer (July 30–31), and qRT-PCR methods were the same as described in another report [27]. The primers used for qRT-PCR analysis of *MFT* are listed in S1 Table.

## Results

### Annual growth pattern in the field

Japanese cedar began to grow in May, exhibited the most active growth in June and July, and continued growing until mid-October (S2 Fig). The initial height of the trees was 47–53 cm, which increased more than 2-fold by December 10 (108–147 cm). The average weekly temperature and day length on May 26, when the trees began growing, was 16.8°C and 14:08, respectively, and on October 15, when the trees stopped growing, these values were 18.4°C and 11:27, respectively. Considering these data, we selected 15°C and 25°C as the temperatures for LT and HT conditions.

### Annual transcriptome dynamics in the field

**Fig 1:**
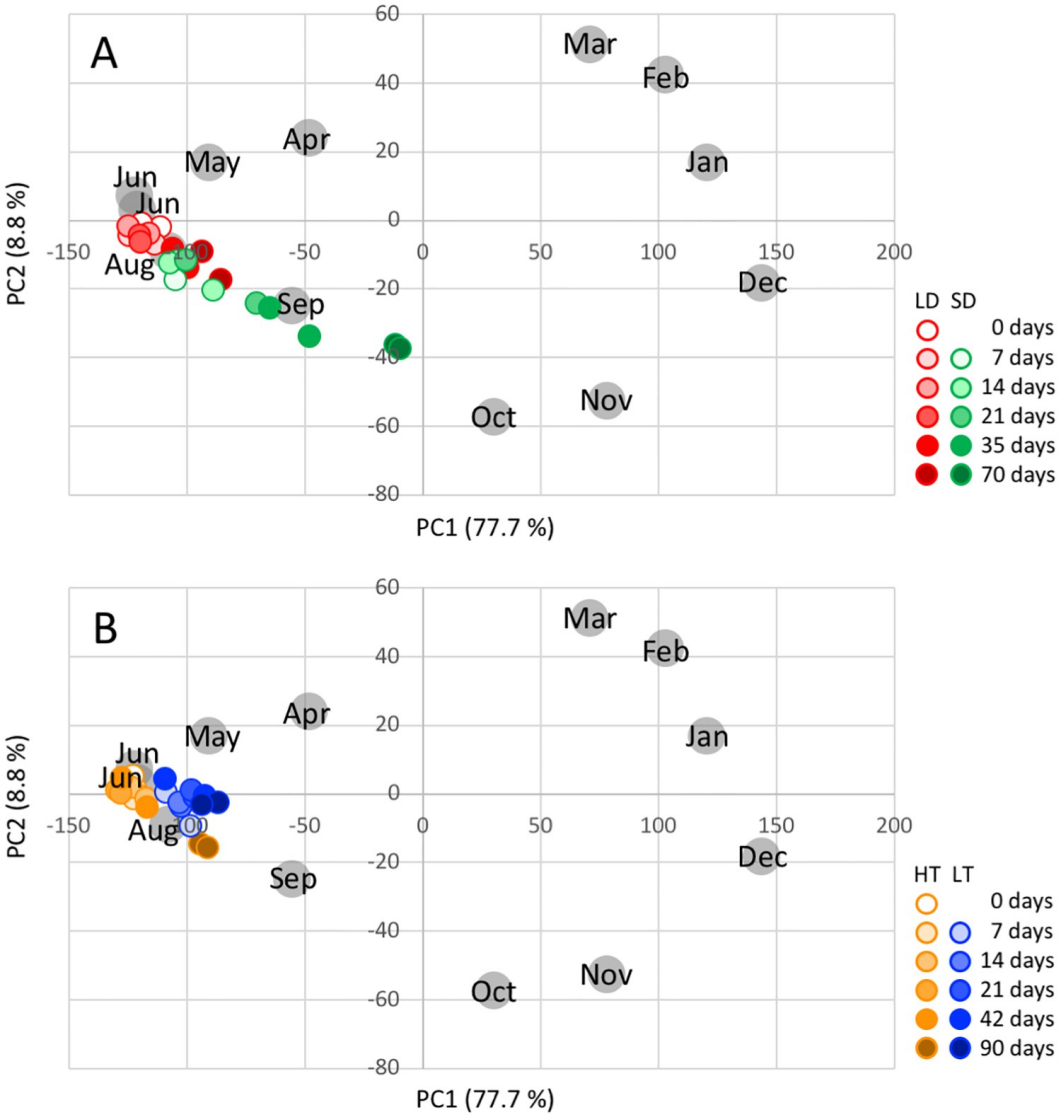
PCA of microarray data. The first two principal components obtained from PCA of annual time series samples of microarray data are shown (gray circles). The seasonal conditions of Japanese cedar grown in a controlled-environment chamber were estimated by plotting microarray data against the PCA results of annual time series samples. (A) Red and green circles indicate samples collected from cuttings grown under LD and SD conditions, respectively. (B) Orange and blue circles indicate samples collected from cuttings grown under HT and LT conditions, respectively.

Annual transcriptome dynamics was clearly demonstrated by PCA (Fig 1). The 12 sets of annual time series data were plotted in a circle. PC1 explained 77.7% of the total variation in gene expression; the December sample had the highest PC1 score (143.5) and the June sample the lowest (−121.0 in 2013 and −122.3 in 2014). PC2 explained 8.8% of the total variation in gene expression; the March sample had the highest PC2 score (51.4) and the October sample the lowest (−57.3).

**Fig 2:**
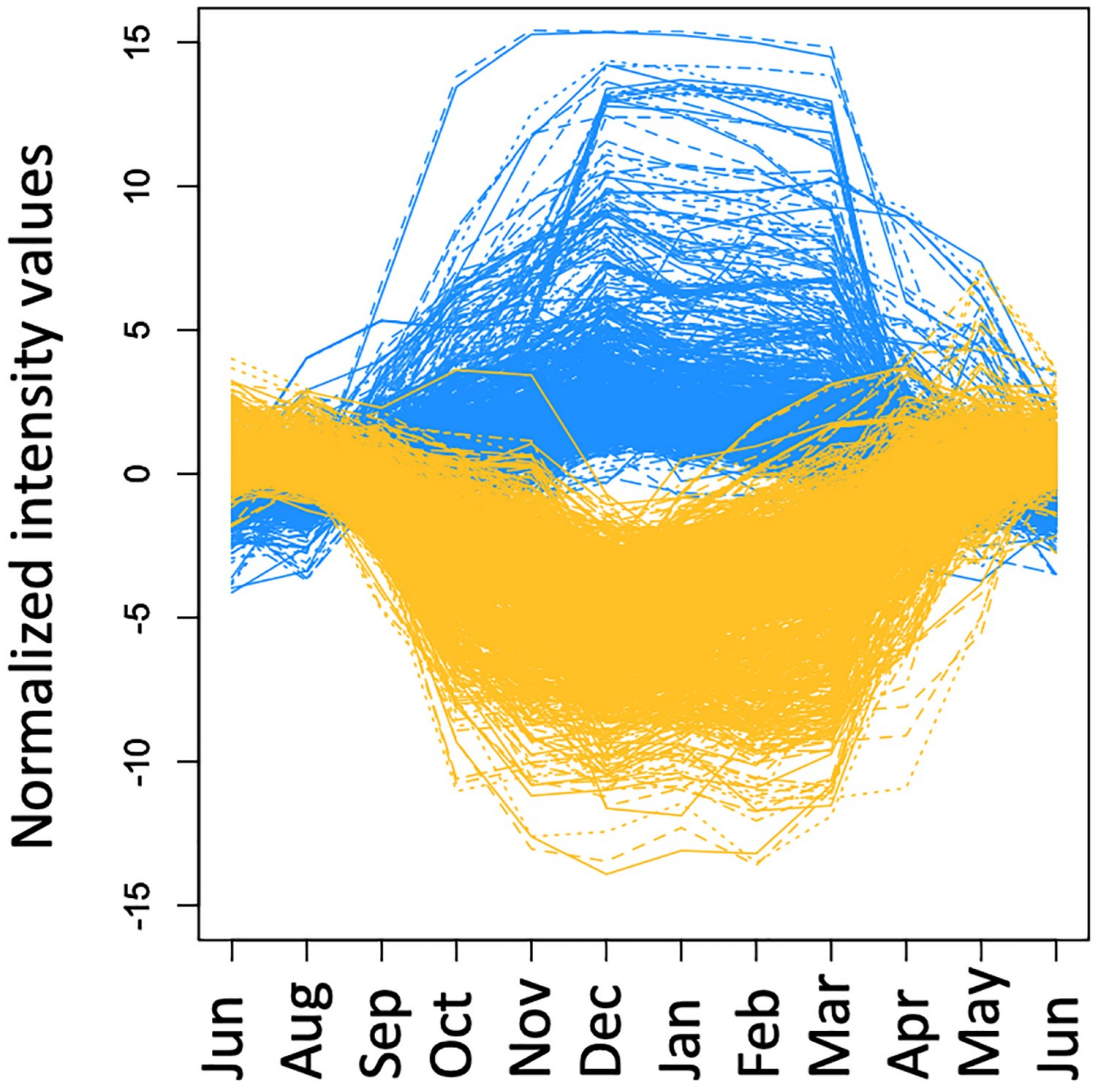
Annual expression pattern of the top-scoring targets of principal component 1. Annual expression patterns of the targets with the top 1,000 positive and negative scores for principal component 1 (blue and orange, respectively). Each line represents the average normalized intensity value of an individual transcript.

Transcripts making the greatest contribution to PC1 were identified according to the absolute value of their component scores. Fig 2 shows the expression profiles of the top 1,000 targets with positive component score values (PC1^+^ targets) and the top 1,000 targets with negative component score values (PC1^−^ targets) (S2 Table). The expression of PC1^+^ targets tended to increase in winter (December) and decrease in summer (June). In contrast, PC1^−^ targets exhibited an opposite expression pattern. The PC1^+^ and PC1^−^ targets were categorized according to major gene ontology (GO) functional categories for biological processes; GO terms of the lowest hierarchy are listed in Table 2. The PC1^−^ targets were enriched in cell wall-related categories, such as ‘cellulose catabolic process’, ‘pectin catabolic process’, ‘plant-type cell wall modification’, ‘xyloglucan metabolic process’, and ‘cell cycle’; the PC1^+^ targets were enriched in genes related to ‘starch metabolic process’ and ‘response to chemical’.

**Table 2:** Functional categories overrepresented among the top-scoring targets of principal component 1.

GO biological process	GO no.	all genes	gene count	expected	fold enrichment	raw P-value	FDR
PC1^−^ targets							
cellulose catabolic process	GO:0030245	8	7	0.74	9.44	1.07E-04	6.55E-03
pectin catabolic process	GO:0045490	15	10	1.39	7.19	1.81E-05	1.32E-03
plant-type cell wall modification	GO:0009827	11	7	1.02	6.86	4.24E-04	2.20E-02
cuticle development	GO:0042335	16	9	1.48	6.07	1.27E-04	7.52E-03
xyloglucan metabolic process	GO:0010411	16	9	1.48	6.07	1.27E-04	7.42E-03
DNA-dependent DNA replication	GO:0006261	15	8	1.39	5.75	3.98E-04	2.09E-02
plant-type secondary cell wall biogenesis	GO:0009834	17	8	1.58	5.07	7.54E-04	3.47E-02
lignin biosynthetic process	GO:0009809	31	14	2.87	4.87	1.20E-05	9.52E-04
cellulose biosynthetic process	GO:0030244	21	9	1.95	4.62	6.04E-04	2.93E-02
microtubule-based process	GO:0007017	38	12	3.52	3.41	7.49E-04	3.48E-02
lipid catabolic process	GO:0016042	61	18	5.66	3.18	8.03E-05	5.18E-03
anatomical structure formation involved in morphogenesis	GO:0048646	55	16	5.1	3.14	2.27E-04	1.28E-02
cell cycle	GO:0007049	123	26	11.41	2.28	4.64E-04	2.38E-02
defense response	GO:0006952	325	50	30.14	1.66	1.10E-03	4.82E-02
PC1^+^ targets							
starch metabolic process	GO:0005982	35	14	3.37	4.15	5.21E-05	4.70E-02
response to chemical	GO:0042221	953	134	91.81	1.46	1.78E-05	4.02E-02

The unique set of *Arabidopsis* gene IDs (e-value <e-5) of the targets of the top 1,000 positive and negative scores for principal component 1 (the analyzed list) was compared to the unique set of *Arabidopsis* gene IDs of 10,439 targets (the reference list) to investigate the overrepresented categories (FDR ≤0.05). Only the lowest categories in the GO hierarchy are listed. The designations “all genes” and “gene count” indicate the number of genes in the reference list and the analyzed list for a particular annotation data category, respectively. The designation “expected” indicates the expected number of genes in the analyzed list based on the reference list. The designation “fold enrichment” indicates the enrichment in the analyzed list based on the reference list. The designations “raw P-value” and “FDR” indicate the probability that the number of genes observed occurred by chance (randomly), as determined based on the reference list.

### Day length- and temperature-responsive targets identified based on experimental conditions

In this study, we perform the experiment twice to identify day length- and then temperature-regulated targets. In the two experiments, Japanese cedar trees were cultivated under LD and HT conditions, in which the environmental conditions consisted of 16 h of light and 8 h of darkness at 25°C, to compare to SD and LT conditions, respectively. Correlation coefficients of the 10,439 targets in the microarray data indicated a degree of correlation of the transcriptome between LD and HT conditions at 0, 7, and 21 days (0.6, 0.6, and 0.7, respectively). The PCA (Fig 1) also demonstrated that the transcriptomes of cuttings from trees grown under LD and HT conditions at 0, 7, and 21 days were similar to the transcriptome of cuttings in June from the field, when trees exhibited the most active growth. These results indicated the reproducibility of tree conditions used in the day length and temperature experimental time series samples. These data also indicated that the experiments accurately represented the day length and temperature responses of Japanese cedar during the growth period.

**Fig 3:**
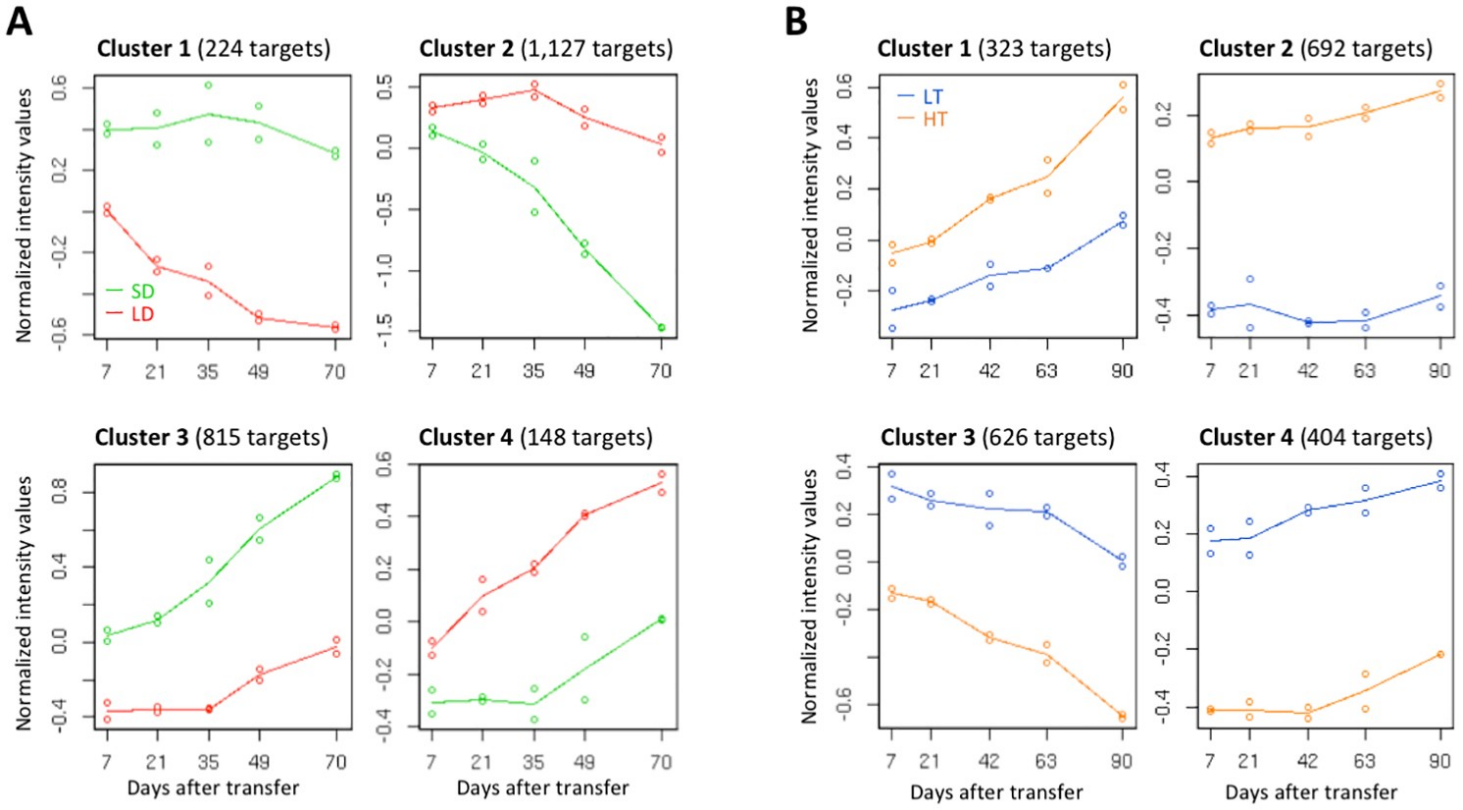
Expression pattern of a cluster of targets regulated by day length and temperature. (A) Median profile of four clusters of SD-regulated targets under LD and SD conditions (red and green, respectively). (B) Median profile of four clusters of LT-regulated targets under HT and LT conditions (orange and blue, respectively).

A total of 2,314 targets (22.2%) exhibited significantly different expression profiles in the time-course experiment between SD and LD conditions (S3 Table). Clustering analyses indicated that 224 targets in Cluster 1 and 815 targets in Cluster 3 tended to be up-regulated under SD conditions, with the expression of Cluster 3 targets increasing over time (Fig 3A). In contrast, a total of 1,127 targets in Cluster 2 and 148 targets in Cluster 4 tended to be down-regulated under SD conditions, with the expression of Cluster 2 targets decreasing over time. Functional categorization based on biological processes indicated significant GO enrichment in each cluster; the GO terms of the lowest hierarchy are listed in Table 3. Cluster 1 consisted of a significantly higher proportion of genes involved in ‘translation’. Cluster 2 consisted of a significantly higher proportion of cell wall- and growth-related categories, such as ‘xylan biosynthetic process’, ‘plant-type secondary cell wall biogenesis’, ‘cellulose biosynthetic process’, ‘pectin metabolic process’, and ‘cell growth’. No statistically significant results were obtained for Clusters 3 and 4.

**Table 3:** Functional categories overrepresented in a cluster of targets regulated by day length and temperature.

GO biological process	GO no.	all genes	gene count	expected	fold enrichment	raw P-value	FDR
**SD-regulated targets**							
** Cluster 1**							
translation	GO:0006412	233	26	6.55	3.97	5.88E-09	8.85E-06
** Cluster 2**							
xylan biosynthetic process	GO:0045492	8	7	1.04	6.75	7.12E-04	4.66E-02
plant-type secondary cell wall biogenesis	GO:0009834	17	13	2.2	5.9	9.97E-06	1.41E-03
cuticle development	GO:0042335	16	11	2.07	5.31	9.67E-05	8.23E-03
microtubule-based process	GO:0007017	38	21	4.92	4.27	1.00E-06	1.74E-04
plant-type cell wall organization	GO:0009664	34	18	4.4	4.09	9.36E-06	1.41E-03
cellulose biosynthetic process	GO:0030244	21	11	2.72	4.04	5.58E-04	3.87E-02
pectin metabolic process	GO:0045488	32	16	4.15	3.86	4.99E-05	5.00E-03
supramolecular fiber organization	GO:0097435	39	16	5.05	3.17	3.04E-04	2.17E-02
phenylpropanoid biosynthetic process	GO:0009699	46	18	5.96	3.02	2.08E-04	1.56E-02
polysaccharide catabolic process	GO:0000272	51	19	6.61	2.88	2.33E-04	1.72E-02
drug catabolic process	GO:0042737	56	19	7.25	2.62	7.06E-04	4.68E-02
cell growth	GO:0016049	126	34	16.32	2.08	3.52E-04	2.48E-02
cellular component morphogenesis	GO:0032989	127	33	16.45	2.01	6.29E-04	4.23E-02
**LT-regulated targets**							
** Cluster 1**							
protein phosphorylation	GO:0006468	170	18	6.31	2.85	1.22E-04	1.90E-02
transcription, DNA-templated	GO:0006351	361	35	13.4	2.61	4.34E-07	6.53E-04
regulation of transcription, DNA-templated	GO:0006355	483	37	17.93	2.06	3.82E-05	6.89E-03
** Cluster 2**							
response to heat	GO:0009408	84	27	7.03	3.84	5.01E-08	2.26E-04
** Cluster 3**							
iron-sulfur cluster assembly	GO:0016226	15	8	1.24	6.43	1.96E-04	3.83E-02
ribosome biogenesis	GO:0042254	127	25	10.53	2.37	2.62E-04	4.72E-02
response to cadmium ion	GO:0046686	173	32	14.35	2.23	8.40E-05	3.79E-02
translation	GO:0006412	233	40	19.32	2.07	5.84E-05	3.76E-02

The unique set of *Arabidopsis* gene IDs (e-value <e-5) of each cluster was compared to the unique set of *Arabidopsis* gene IDs of 10,439 targets to investigate the overrepresented categories (FDR ≤0.05). Only the lowest categories in the GO hierarchy are listed. There were no overrepresented categories for Cluster 3 or Cluster 4 of SD-regulated targets or for Cluster 4 of LT-regulated targets. The designations “all genes” and “gene count” indicate the number of genes in the reference list and the analyzed list for a particular annotation data category, respectively. The designation “expected” indicates the expected number of genes in the analyzed list based on the reference list. The designation “fold enrichment” indicates the enrichment in the analyzed list based on the reference list. The designations “raw P-value” and “FDR” indicate the probability that the number of genes observed occurred by chance (randomly), as determined based on the reference list.

The same statistical analyses were carried out to identify LT-regulated targets using the time series microarray data for LT and HT conditions. A total of 2,045 targets (19.6%) exhibited significantly different expression profiles under LT and HT conditions (S3 Table). Of these targets, 323 targets in Cluster 1 and 692 targets in Cluster 2 tended to be down-regulated under LT conditions, and the expression of targets in Cluster 1 increased over time (Fig 3B). In contrast, 626 targets in Cluster 3 and 404 targets in Cluster 4 tended to be up-regulated under LT conditions, and the expression of Cluster 3 targets decreased over time. GO analyses indicated that Cluster 1 consisted of a significantly higher proportion of genes involved in ‘protein phosphorylation’, ‘transcription, DNA-templated’, and ‘regulation of transcription, DNA-templated’, whereas Cluster 2 consisted of a significantly higher proportion of genes involved in ‘response to heat’, including putative genes encoding heat shock proteins (Table 3). Cluster 3, which contained LT- up-regulated targets, contained a significantly higher proportion of genes involved in ‘iron-sulfur cluster assembly’, ‘ribosome biogenesis’, ‘response to cadmium ion’, and ‘translation’. No statistically significant results were obtained for Cluster 4.

### Contribution of SD- and LT-regulated targets to annual transcriptome dynamics

To determine seasonal differences at the transcriptome level in cuttings grown under SD and LD conditions, microarray data obtained from cuttings of trees grown in a controlled-environment chamber were compared to the PCA results for annual series under natural conditions (Fig 1A). Before transfer to SD conditions (0 days), the transcriptome of the cuttings was similar to the transcriptome observed in June. The PC1 scores increased and PC2 scores decreased over time, and the transcriptome after 70 days under SD conditions was similar to the transcriptome in autumn, between September and October. In contrast, the transcriptome of cuttings from trees grown under LT conditions exhibited a low PC1 score during the 90 days of the experiment and was similar to the transcriptome in August (Fig 1B).

**Fig 4:**
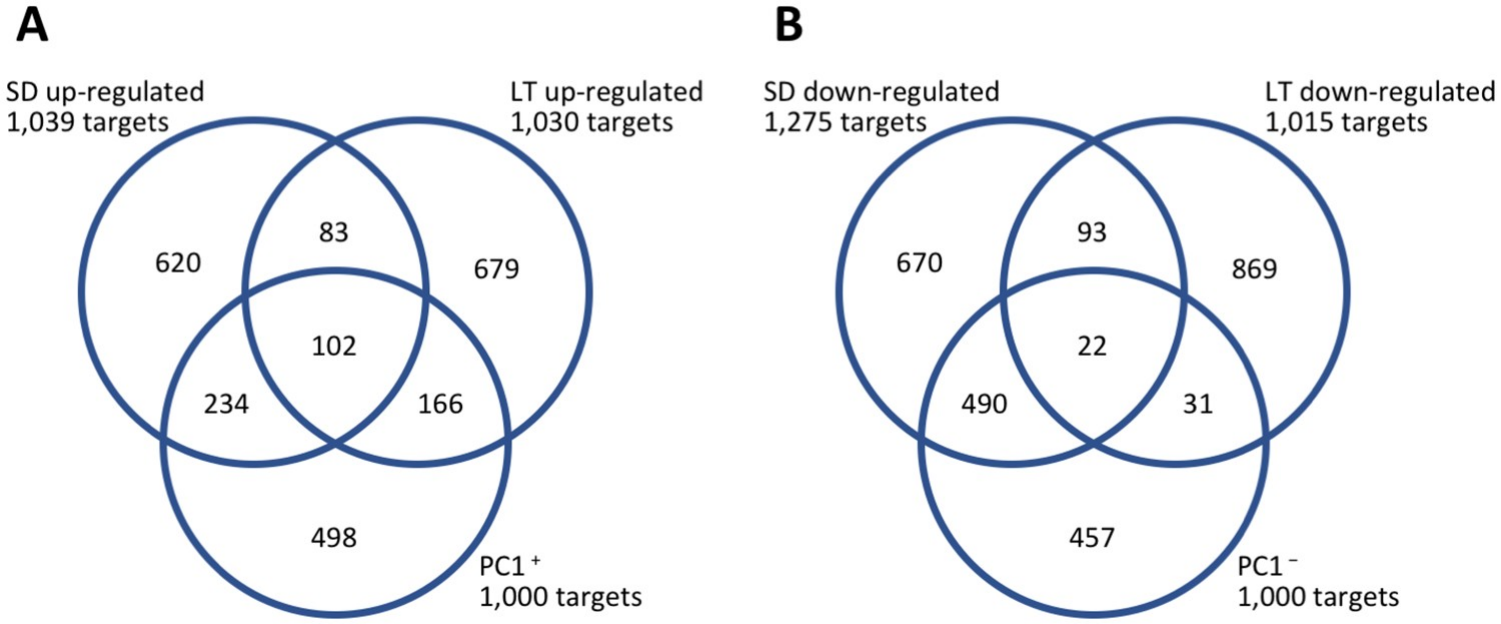
Comparison of genes regulated under SD and LT conditions and top 1,000 positive and negative scoring targets of principal component 1. Expression of targets with positive scores for principal component 1 increased in winter, and expression of targets with negative scores increased in summer; refer to Fig 2.

The percentages of SD- and LT-regulated targets within the PC1^+^ and PC1^−^ groups were also calculated to investigate their contribution to annual transcriptome dynamics (Fig 4). Overall, PC1^+^ targets included 336 targets up-regulated under SD conditions and 268 up-regulated under LT conditions (50.2% of total), whereas PC1^−^ included 512 targets down-regulated under SD conditions and 53 down-regulated under LT conditions (54.3% of total).

**Fig 5:**
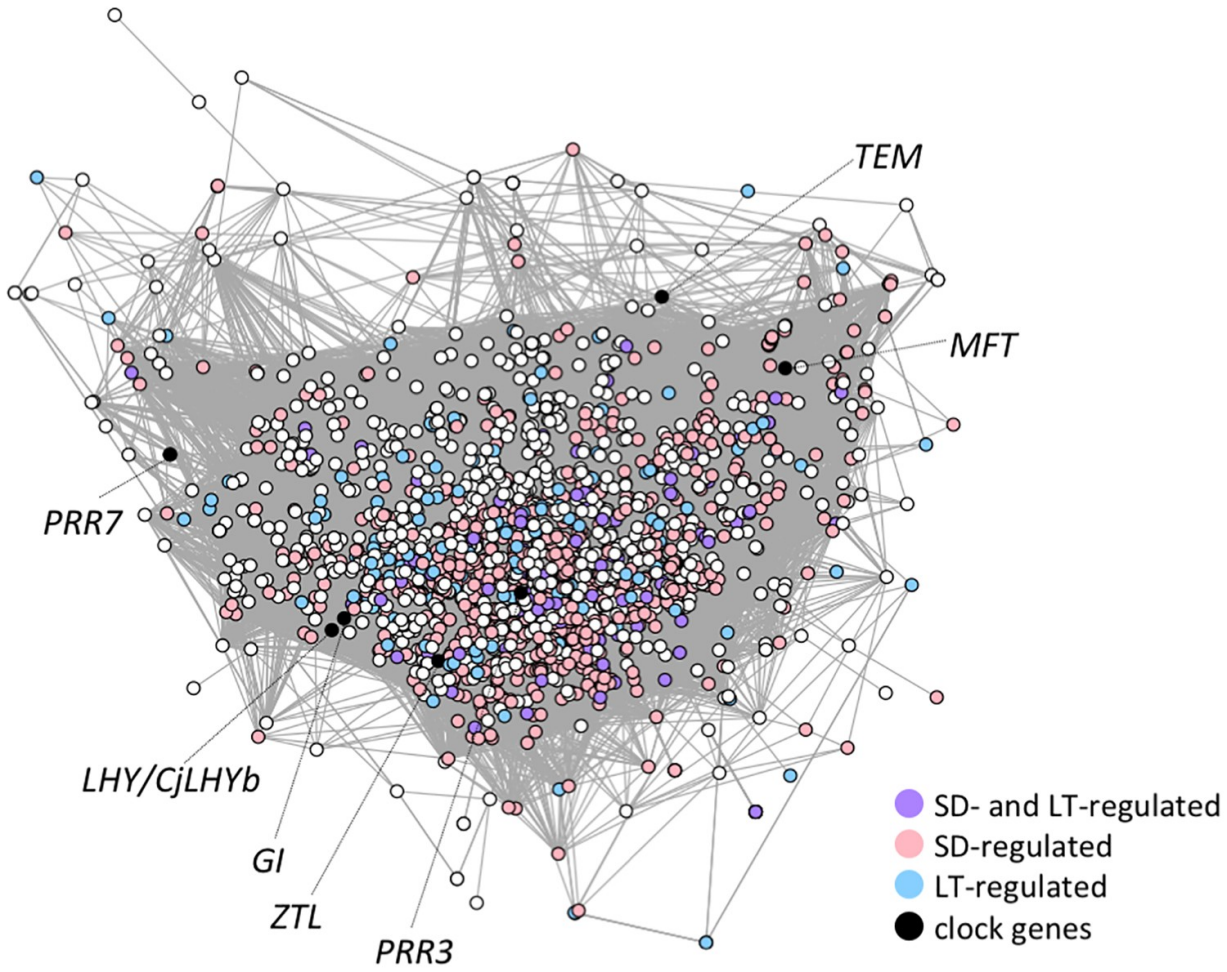
Co-expression gene network of annual transcriptome dynamics. The gene network was estimated based on correlation coefficients of the expression value between genes using the microarray data of annual time series samples, and 1,953 targets were connected. Dot colors indicate the results of experimental time series samples; “SD-regulated” indicates targets that were up- or down-regulated under SD conditions, “LT-regulated” indicates targets that were up- or down-regulated under LT conditions, and “SD- and LT-regulated” indicates targets that were up- or down-regulated under both SD and LT conditions. This result demonstrated a close relationship between SD- and LT-regulated genes in the annual transcriptome dynamics.

Gene co-expression network analyses of the 1,976 targets for which the gene pairs exhibited highly correlated expression patterns revealed that 1,953 of the targets formed a large cluster (Fig 5). These 1,953 clustered targets included 892 targets (45.7%) of SD up- and down-regulated targets (SD-regulated targets) and 256 targets (13.1%) of LT up- and down-regulated targets (LT-regulated targets); a total of 117 targets (6.0%) were both SD- and LT-regulated. The network analysis indicated a mixture of SD- and LT-regulated targets, indicating that these genes contribute significantly to annual transcriptome dynamics.

### Effects of day length and temperature on genes related to growth and starch metabolism

PC1^−^ targets that tended to exhibit increased expression in summer (Fig 2) also exhibited significant enrichment of GO terms related to growth (Table 2). The effects of day length and temperature on the expression of genes in growth-related categories were investigated by analyzing the ratio of targets down-regulated under SD conditions to those down-regulated under LT conditions. The GO term ‘cellulose biosynthetic process’ was associated with 11 PC1^−^ targets, all of which were down-regulated under SD conditions (Table 4). The terms ‘pectin catabolic process’, ‘plant-type cell wall modification’, and ‘lignin biosynthetic process’ were associated with 13, 17, and 35 PC1^−^ targets, respectively, and 11 (84.6%), 13 (76.5%), and 20 (57.1%) of these targets were down-regulated under SD conditions. In contrast, only two targets in these four categories were down-regulated under LT conditions, both of which were genes encoding S-adenosylmethionine synthetase family protein (*MTO3*) in the category ‘lignin biosynthetic process’.

**Table 4:** Cell wall-related genes and effects of day length and temperature.

sequence ID	*Arabidopsis* ID	e-value	gene	description	day length		temperature	
					up/down*	cluster	up/down*	cluster
**pectin catabolic process**								
reCj17068ex_ne:----:isotig17035	AT1G67750	1.32E-112	-	Pectate lyase family protein	down	2	-	-
reCj12911ex_ne:---W:isotig12878	AT2G36710	8.95E-93	-	Pectin lyase-like superfamily protein	down	2	-	-
reCj09874ex_ne:-LSW:isotig09841	AT3G43270	1.68E-147	-	Plant invertase/pectin methylesterase inhibitor superfamily	down	2	-	-
reCj09987ex_ne:MLSW:isotig09954	AT3G49220	0	-	Plant invertase/pectin methylesterase inhibitor superfamily	down	2	-	-
reCj11252ex_ne:-L-W:isotig11219	AT4G13710	0	-	Pectin lyase-like superfamily protein	down	2	-	-
reCj12066ex_ne:---W:isotig12033	AT4G13710	0	-	Pectin lyase-like superfamily protein	down	2	-	-
reCj11253ex_ne:-L--:isotig11220	AT4G24780	0	-	Pectin lyase-like superfamily protein	down	2	-	-
reCj18816ex_ne:M---:isotig18783	AT4G24780	4.41E-149	-	Pectin lyase-like superfamily protein	down	2	-	-
reCj10947ex_ne:MLSW:isotig10914	AT4G24780	0	-	Pectin lyase-like superfamily protein	down	2	-	-
reCj15431ex_ne:----:isotig15398	AT5G47500	2.95E-144	-	Pectin lyase-like superfamily protein	down	2	-	-
reCj09812ex_ne:-LS-:isotig09779	AT5G53370	2.12E-161	*PMEPCRF*	pectin methylesterase PCR fragment F	down	2	-	-
reCj12181ex_ne:-L--:isotig12148	AT2G45220	1.26E-108	-	Plant invertase/pectin methylesterase inhibitor superfamily	-	-	-	-
reCj05279ex_ne:MLS-:isotig05253	AT4G02330	2.28E-160	*PMEPCRB*	Plant invertase/pectin methylesterase inhibitor superfamily	-	-	-	-
**plant-type cell wall modification**			-					
reCj14972ex_ne:MLSW:isotig14939	AT1G26770	2.32E-124	*EXP10*	expansin A10	down	2	-	-
reCj14461ex_ne:-L-W:isotig14428	AT1G26770	8.03E-141	*EXP10*	expansin A10	down	2	-	-
reCj14263ex_ne:-L--:isotig14230	AT1G69530	9.24E-132	*EXP1*	expansin A1	down	2	-	-
reCj14486ex_ne:MLSW:isotig14453	AT1G69530	5.89E-133	*EXP1*	expansin A1	down	2	-	-
reCj13170ex_ne:MLSW:isotig13137	AT2G39700	7.22E-151	*EXP4*	expansin A4	down	2	-	-
reCj15212ex_ne:--S-:isotig15179	AT2G40610	6.96E-138	*EXP8*	expansin A8	down	2	-	-
reCj15134ex_ne:MLS-:isotig15101	AT2G40610	4.71E-134	*EXP8*	expansin A8	down	2	-	-
reCj21639ex_ne:-L--:isotig21606	AT2G40610	3.20E-79	*EXP8*	expansin A8	down	2	-	-
reCj15071ex_ne:M---:isotig15038	AT2G40610	6.34E-140	*EXP8*	expansin A8	down	2	-	-
reCj15348ex_ne:MLS-:isotig15315	AT2G40610	1.20E-134	*EXP8*	expansin A8	down	2	-	-
reCj03290ex_ne:-LSW:isotig03265	AT4G28250	4.07E-115	*EXPB3*	expansin B3	down	2	-	-
reCj03291ex_ne:-LSW:isotig03266	AT4G28250	1.61E-120	*EXPB3*	expansin B3	down	2	-	-
reCj15940ex_ne:----:isotig15907	AT4G28250	5.28E-99	*EXPB3*	expansin B3	down	2	-	-
reCj20135ex_ne:----:isotig20102	AT1G10550	7.14E-54	*XET*	xyloglucan:xyloglucosyl transferase 33	-	-	-	-
reCj05655ex_ne:ML--:isotig05629	AT1G69530	2.83E-133	*EXP1*	expansin A1	-	-	-	-
reCj15237ex_ne:-L--:isotig15204	AT4G34980	5.74E-77	-	subtilisin-like serine protease 2	-	-	-	-
reCj14335ex_ne:-L--:isotig14302	AT4G34980	7.28E-70	-	subtilisin-like serine protease 2	-	-	-	-
**cellulose biosynthetic process**								
reCj04931ex_ne:MLSW:isotig04905	AT1G05850	6.24E-138	*POM1*	Chitinase family protein	down	2	-	-
reCj14318ex_ne:ML-W:isotig14285	AT1G05850	1.37E-102	*POM1*	Chitinase family protein	down	2	-	-
reCj07613ex_ne:--SW:isotig07580	AT2G22125	0	-	binding	down	2	-	-
reCj11007ex_ne:MLSW:isotig10974	AT3G02230	0	*RGP1*	reversibly glycosylated polypeptide 1	down	2	-	-
reCj12645ex_ne:----:isotig12612	AT3G12060	1.88E-119	-	Plant protein of unknown function (DUF828)	down	2	-	-
reCj11124ex_ne:-L-W:isotig11091	AT3G12060	1.89E-101	-	Plant protein of unknown function (DUF828)	down	2	-	-
reCj07972ex_ne:-L-W:isotig07939	AT4G18780	0	*CESA8*	cellulose synthase family protein	down	2	-	-
reCj07549ex_ne:MLSW:isotig07516	AT4G39350	0	*CESA2*	cellulose synthase A2	down	2	-	-
reCj07710ex_ne:-L-W:isotig07677	AT5G17420	0	*IRX3*	Cellulose synthase family protein	down	2	-	-
reCj07868ex_ne:-L-W:isotig07835	AT5G44030	0	*CESA4*	cellulose synthase A4	down	2	-	-
reCj08597ex_ne:MLSW:isotig08564	AT5G49720	0	*GH9A1*	glycosyl hydrolase 9A1	down	2	-	-
**lignin biosynthetic process**			-					
reCj04931ex_ne:MLSW:isotig04905	AT1G05850	6.24E-138	*POM1*	Chitinase family protein	down	2	-	-
reCj14318ex_ne:ML-W:isotig14285	AT1G05850	1.37E-102	*POM1*	Chitinase family protein	down	2	-	-
reCj12615ex_ne:ML-W:isotig12582	AT1G52760	8.02E-157	-	lysophospholipase 2	down	2	-	-
reCj02719ex_ne:MLS-:isotig02697	AT1G77520	2.68E-53	-	O-methyltransferase family protein	down	2	-	-
reCj12024ex_ne:ML-W:isotig11991	AT3G17390	0	*MTO3*	S-adenosylmethionine synthetase family protein	down	2	-	-
reCj01185ex_ne:ML-W:isotig01163	AT3G17390	0	*MTO3*	S-adenosylmethionine synthetase family protein	down	2	-	-
reCj01188ex_ne:ML-W:isotig01166	AT3G17390	0	*MTO3*	S-adenosylmethionine synthetase family protein	down	2	down	2
reCj01189ex_ne:ML-W:isotig01167	AT3G17390	0	*MTO3*	S-adenosylmethionine synthetase family protein	down	2	down	2
reCj13644ex_ne:-LS-:isotig13611	AT4G30470	5.24E-96	-	NAD(P)-binding Rossmann-fold superfamily protein	down	2	-	-
reCj17236ex_ne:-L-W:isotig17203	AT4G34050	5.28E-13	-	S-adenosyl-L-methionine-dependent methyltransferases superfamily protein	down	2	-	-
reCj16880ex_ne:---W:isotig16847	AT4G34050	5.64E-16	*CCoAOMT1*	S-adenosyl-L-methionine-dependent methyltransferases superfamily protein	down	2	-	-
reCj04773ex_ne:-L-W:isotig04747	AT5G03260	0	*LAC11*	laccase 11	down	2	-	-
reCj09794ex_ne:-LSW:isotig09761	AT5G03260	1.72E-157	*LAC11*	laccase 11	down	2	-	-
reCj05268ex_ne:-LS-:isotig05242	AT5G05340	4.67E-132	-	Peroxidase superfamily protein	down	2	-	-
reCj05267ex_ne:MLS-:isotig05241	AT5G05340	1.98E-131	-	Peroxidase superfamily protein	down	2	-	-
reCj16928ex_ne:MLSW:isotig16895	AT5G05340	1.04E-118	-	Peroxidase superfamily protein	down	2	-	-
reCj15497ex_ne:M---:isotig15464	AT5G05340	7.37E-124	-	Peroxidase superfamily protein	down	2	-	-
reCj14489ex_ne:--S-:isotig14456	AT5G05340	4.60E-141	-	Peroxidase superfamily protein	down	2	-	-
reCj16106ex_ne:M---:isotig16073	AT5G54160	5.65E-62	*OMT1*	O-methyltransferase 1	down	2	-	-
reCj05373ex_ne:MLSW:isotig05347	AT5G66390	1.37E-126	-	Peroxidase superfamily protein	down	2	-	-
reCj00680ex_ne:----:isotig00658	AT1G67980	1.14E-14	*CCOAMT*	caffeoyl-CoA 3-O-methyltransferase	-	-	-	-
reCj02720ex_ne:----:isotig02698	AT1G77520	7.71E-53	-	O-methyltransferase family protein	-	-	-	-
reCj02721ex_ne:MLS-:isotig02699	AT1G77520	1.08E-53	-	O-methyltransferase family protein	-	-	-	-
reCj12563ex_ne:-L--:isotig12530	AT4G01070	8.35E-104	*UGT72B1*	UDP-Glycosyltransferase superfamily protein	-	-	-	-
reCj00676ex_ne:---W:isotig00654	AT4G34050	1.80E-39	*CCoAOMT1*	S-adenosyl-L-methionine-dependent methyltransferases superfamily protein	-	-	-	-
reCj00675ex_ne:--S-:isotig00653	AT4G34050	1.02E-35	*CCoAOMT1*	S-adenosyl-L-methionine-dependent methyltransferases superfamily protein	-	-	-	-
reCj00674ex_ne:--S-:isotig00652	AT4G34050	5.16E-44	*CCoAOMT1*	S-adenosyl-L-methionine-dependent methyltransferases superfamily protein	-	-	-	-
reCj00677ex_ne:M-SW:isotig00655	AT4G34050	4.64E-50	*CCoAOMT1*	S-adenosyl-L-methionine-dependent methyltransferases superfamily protein	-	-	-	-
reCj00678ex_ne:M-SW:isotig00656	AT4G34050	3.61E-39	*CCoAOMT1*	S-adenosyl-L-methionine-dependent methyltransferases superfamily protein	-	-	-	-
reCj11392ex_ne:M--W:isotig11359	AT4G39330	6.26E-128	*CAD9*	cinnamyl alcohol dehydrogenase 9	-	-	-	-
reCj14819ex_ne:-LS-:isotig14786	AT5G05340	2.10E-122	-	Peroxidase superfamily protein	-	-	-	-
reCj15586ex_ne:----:isotig15553	AT5G54160	4.23E-83	*OMT1*	O-methyltransferase 1	-	-	-	-
reCj05195ex_ne:MLSW:isotig05169	AT5G54160	1.08E-79	*OMT1*	O-methyltransferase 1	-	-	-	-
reCj16432ex_ne:-L--:isotig16399	AT5G54160	3.25E-55	*OMT1*	O-methyltransferase 1	-	-	-	-
reCj09808ex_ne:-L-W:isotig09775	AT5G60020	0	*LAC17*	laccase 17	-	-	-	-

The targets of GO categories ‘pectin catabolic process’, ‘plant-type cell wall modification’, ‘cellulose biosynthetic process’, and ‘lignin biosynthetic process’, which constituted the top 1,000 negative scores for principal component 1, are listed.

*Indicates up- or down-regulation under SD (day length) or LT (temperature) conditions.

**Fig 6:**
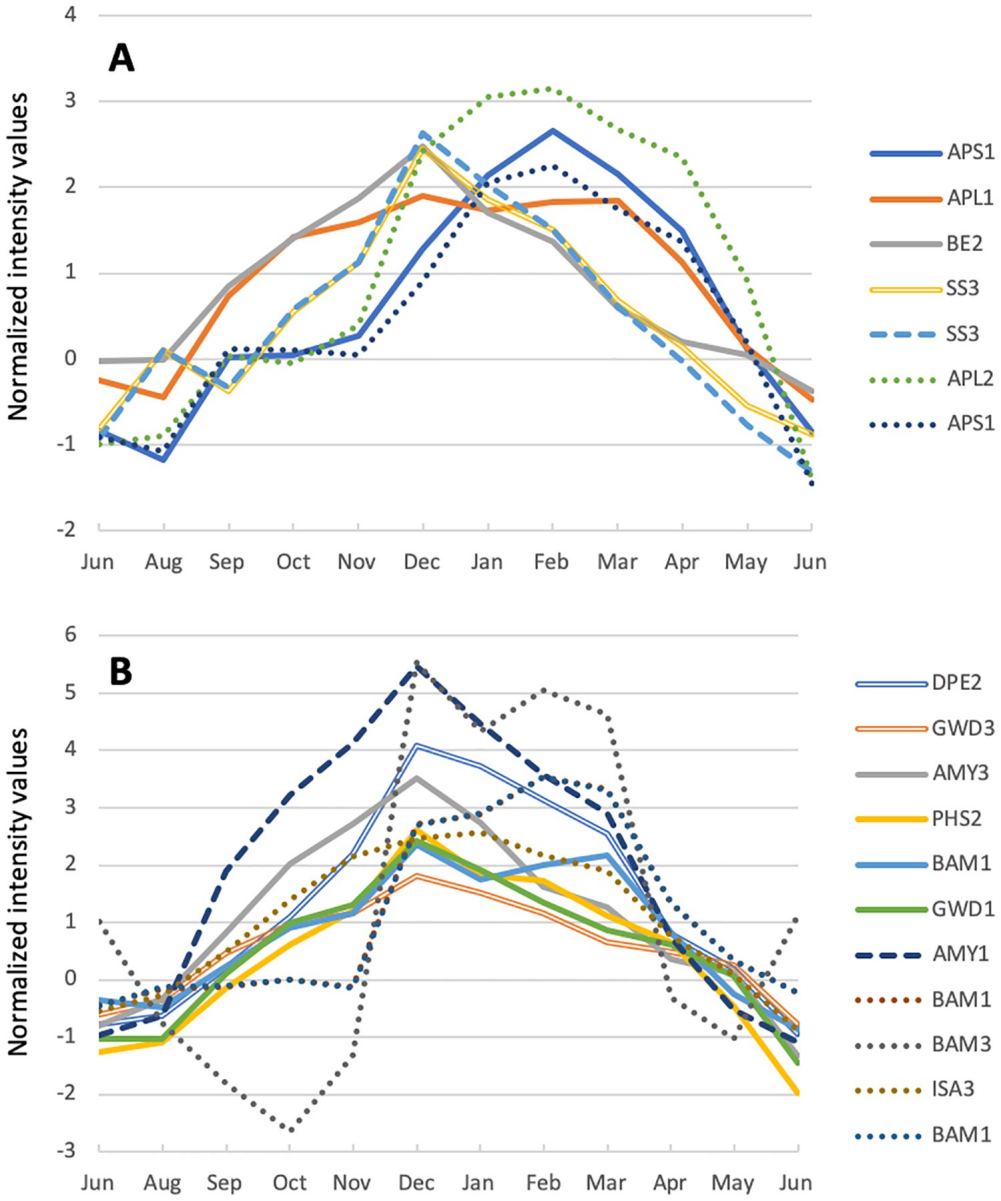
Annual expression patterns of starch-related genes. Each line represents the average normalized intensity value of an individual transcript of a gene involved in starch synthesis (A) or starch degradation (B), as listed in Table 5. Broken lines and solid lines indicate transcripts up-regulated by SD and LT conditions, respectively. Double lines indicate transcripts up-regulated by both SD and LT conditions. Dotted lines indicate transcripts not up-regulated by either SD or LT conditions in this study.

The PC1^+^ targets exhibited significant enrichment in the term ‘starch metabolic process’ (Table 2). A total of 42 targets related to starch breakdown and starch synthesis were identified that were described in a previous report from a study involving *Arabidopsis* [34], and 18 of these targets (42.9%) were PC1^+^, and their expression tended to increase in winter (Fig 6, Table 5). Of the seven starch synthesis-related gene targets, two were up-regulated under SD conditions and three under LT conditions. The expression of two other targets, ADPGLC-PPase large subunit (*APL2*) and ADP glucose pyrophosphorylase 1 (*APS1*), which was not up-regulated under SD or LT conditions, peaked in February and then decreased dramatically from April to May (Fig 6A). Of the 11 starch breakdown-related gene targets, three were up-regulated under SD conditions and six under LT conditions, and their expression peaked in December (Fig 6B, Table 5). The expression of other targets that were not up-regulated under either SD or LT conditions tended to be high from December to March (Fig 6B).

**Table 5:** Effects of day length and temperature on genes related to starch synthesis and degradation.

sequence ID	*Arabidopsis* ID	e-value	gene	description	day length		temperature	
					up/down*	cluster	up/down*	cluster
**starch synthesis-related genes**								
reCj13814ex_ne:-LSW:isotig13781	AT1G11720	1.34E-97	*SS3/SSIII*	starch synthase 3	up	1	down	2
reCj08308ex_ne:--SW:isotig08275	AT1G11720	1.20E-126	*SS3/SSIII*	starch synthase 3	up	3	-	-
reCj10136ex_ne:MLSW:isotig10103	AT5G48300	0	*APS1/ADG1*	ADP glucose pyrophosphorylase 1	-	-	up	3
reCj07437ex_ne:-L--:isotig07411	AT5G19220	0	*APL1/ADG2*	ADP glucose pyrophosphorylase large subunit 1	-	-	up	3
reCj08352ex_ne:-LSW:isotig08319	AT5G03650	0	*BE2*	starch branching enzyme 2.2	-	-	up	3
reCj09314ex_ne:MLSW:isotig09281	AT1G27680	0	*APL2*	ADPGLC-PPase large subunit	-	-	-	-
reCj16413ex_ne:---W:isotig16380	AT5G48300	1.87E-10	*APS1/ADG1*	ADP glucose pyrophosphorylase 1	-	-	-	-
**starch breakdown-related genes**								
reCj11660ex_ne:-LSW:isotig11627	AT4G25000	8.77E-141	*AMY1*	alpha-amylase-like	up	3	-	-
reCj07772ex_ne:M-SW:isotig07739	AT2G40840	0	*DPE2*	disproportionating enzyme 2	up	3	up	4
reCj07644ex_ne:MLSW:isotig07611	AT5G26570	0	*PWD*, *GWD3*	catalytics;carbohydrate kinases;phosphoglucan	up	3	up	4
reCj07821ex_ne:-LSW:isotig07788	AT1G69830	0	*AMY3*	alpha-amylase-like 3	-	-	up	4
reCj07891ex_ne:MLSW:isotig07858	AT3G46970	0	*PHS2*	alpha-glucan phosphorylase 2	-	-	up	4
reCj08867ex_ne:MLSW:isotig08834	AT3G23920	0	*BAM1 (BMY7 / TR-BMY)*	beta-amylase 1	-	-	up	4
reCj07552ex_ne:MLSW:isotig07519	AT1G10760	0	*GWD1*, *SEX1*	Pyruvate phosphate dikinase	-	-	up	4
reCj03097ex_ne:MLSW:isotig03072	AT3G23920	0	*BAM1 (BMY7 / TR-BMY)*	beta-amylase 1	down	4	-	-
reCj10358ex_ne:-LSW:isotig10325	AT4G17090	0	*BAM3 (BMY8 / ctBMY)*	chloroplast beta-amylase	down	2	-	-
reCj08227ex_ne:-LSW:isotig08194	AT4G09020	0	*ISA3*	isoamylase 3	-	-	-	-
reCj03098ex_ne:-LSW:isotig03073	AT3G23920	0	*BAM1 (BMY7 / TR-BMY)*	beta-amylase 1	-	-	-	-

Shown are target starch synthesis- and degradation-related genes from a previous report [34] that were included in the top 1,000 positive scores for principal component 1.

*Indicates up- or down-regulation under SD (day length) or LT (temperature) conditions.

## Discussion

### Seasonal transcriptome dynamics and contribution of day length- and temperature-regulated genes in Japanese cedar

Japanese cedar, which is as an indeterminate species [4], exhibited continuous growth under LD and HT conditions in the controlled-environment chamber (S4 Fig) and continued growing from May through the middle of October, when the day length and temperature declined (day length 11:27, temperature 18.4°C) (S2 Fig). Whereas the growth of determinate species is controlled endogenously and day length and temperature play minor roles in growth cessation, the growth of indeterminate species may be more susceptible to both of these environmental factors [4, 16, 22].

Our microarray data demonstrated dramatic changes in transcripts during the year. To the best of our knowledge, this is the first report describing annual transcriptome dynamics in a gymnosperm indeterminate species. Based on comparisons of all combinations of the 12 sets of annual time series data (Table 1), 28.8% of transcripts (3,004 targets) exhibited expression differences ≥5-fold in at least one combination. PCA of the microarray data demonstrated continuous changes in the transcriptome throughout the year that appeared as an annual circle when plotted (Fig 1). Among the 12 samples acquired during the year, the December sample exhibited the highest and the June sample the lowest PC1 score, which explained 77.7% of the total variation in gene expression. As day length was greatest in June and least in December (S1 Fig), the PCA results suggest that day length plays an important role in annual transcriptome dynamics.

By analyzing trees grown under different experimental conditions (Table 1), we identified 2,314 SD-regulated targets and 2,045 LT-regulated targets, each of which accounted for approximately 20% of the 10,439 targets analyzed. Interestingly, only 429 targets were regulated by both SD and LT. A previous study using transgenic plants incapable of sensing short days indicated that short day and low temperature conditions induced cold acclimation by independent pathways in *Populus* [35]. Our data also appear to suggest these environmental factors are associated with different regulatory mechanisms. Growth cessation and bud formation were induced by SD conditions, whereas growth cessation and development of reddish-brown needles were induced by LT, in agreement with previous reports on Japanese cedar and other coniferous species (S4 Fig) [3, 5, 36–38]. These traits enable the trees to survive in harsh winter environments.

The expression patterns of SD- and LT-regulated targets under natural conditions revealed their contribution to annual transcriptome dynamics. In this study, adult trees (15 to 16 years old) were used as annual time series samples, and juvenile trees (1 year old) were used as experimental time series samples due to limitations in chamber size. Although differences in phenology between juvenile and adult trees have not been investigated in Japanese cedar, it has been reported in temperate deciduous forest tree species [39]. The genes that exhibited annual expression in adult trees in the field and regulated in juvenile trees under environmental conditions may be genes universally regulated by day length and temperature regardless of tree age. The PC1^−^ and PC1^+^ targets that exhibited dramatic annual cycles in expression are hypothesized to be primarily affected by day length and temperature under field conditions. We found that SD- and LT-regulated targets comprised more than half of the PC1^−^ and PC1^+^ targets (Figs 2 and 4). This means that these targets were affected by either day length or temperature and that interactions between day length and temperature were not necessary to exhibit annual expression. Specifically, 336 targets were up-regulated by SD without LT and 268 targets were up-regulated by LT without SD within the PC1^+^ targets (Fig 4A), and 512 targets were down-regulated by SD without LT and 53 targets were down-regulated by LT without SD within the PC1^−^ targets (Fig 4B). The remaining targets could primarily include genes that were up-regulated only by the interaction between day length and temperature.

The largest cluster in the annual transcriptome dynamics gene co-expression network consisted of 97.7% of the 2,000 analyzed targets, with the SD- and LT-regulated targets located in close proximity in the network (Fig 5). These observations indicate that annual transcriptome dynamics may be regulated by both the interaction between day length and temperature and the respective effects of day length and temperature. Although day length and temperature appear to regulate different genes, there is a close relationship between day length- and temperature-related genes in annual transcriptome dynamics.

PCA of the microarray data for trees grown in the controlled-environment chamber also indicated that SD-regulated genes contribute significantly to transition to the dormant state. The PC1 score of the transcriptome increased and the PC2 score decreased over time for SD-regulated targets, indicating that the transcriptome was shifted toward a state of dormancy by SD conditions (Fig 1A). In contrast, the LT-regulated targets exhibited a low PC1 score that over the course of the 90-day experiment was similar to the transcriptome in June and August, when Japanese cedar exhibits maximal growth (Fig 1B). These data demonstrated that SD-regulated genes contribute significantly to the transition to initial dormancy at the transcript level. Although growth cessation was affected by both SD and LT conditions (S4 Fig), LT-regulated targets contributed less to the transition to dormancy at the transcript level.

### Growth-related genes are down-regulated by short-day rather than low-temperature conditions

Although growth was suppressed under both SD and LT conditions, the down-regulation of growth-related genes in autumn may be mostly attributable to day length. The genes in cell wall-related GO categories, such as ‘pectin catabolic process’, ‘plant-type cell wall modification’, ‘lignin biosynthetic process’, and ‘cellulose biosynthetic process’, constituted a significantly higher proportion of PC1^−^ targets, the expression of which increased during the growth period (Table 2). Most of the related genes were classified in Cluster 2 of the SD-regulated targets (Table 4), the expression of which decreased under SD conditions (Fig 3A).

Pectin is a structural heteropolysaccharide present in the primary cell walls of plants and contributes to complex physiologic processes such as cell growth and differentiation [40]. Most genes categorized as ‘pectin catabolic process’ were down-regulated under SD conditions (84.6%), including genes encoding pectin lyase family proteins and the plant invertase/pectin methylesterase inhibitor superfamily (Table 4).

Expansins promote cell wall loosening and extension and are encoded by a superfamily of genes [41]. The ‘plant-type cell wall modification’ category included 14 expansin gene targets, 13 of which were down-regulated under SD conditions. In addition, all of the genes related to ‘cellulose biosynthetic process’ and 57.1% of those related to ‘lignin biosynthetic process’ were down-regulated under SD conditions (Table 4). Shortened day length in autumn may repress expression of these genes related to cell growth, and may even block cell growth, leading to growth cessation. In contrast, only two genes encoding S-adenosylmethionine synthetase family proteins within these four GO categories were down-regulated under LT conditions (Table 4). Growth suppression under LT conditions may involve a different mechanism compared with SD conditions.

### Only a few starch-related genes are induced by SD conditions in Japanese cedar

The expression of starch-related genes may induce starch synthesis or breakdown to enhance cold and frost tolerance and contribute energy for bud breaking and shoot growth in spring [42–44]. In this study, the expression of 18 of 42 analyzed genes involved in starch synthesis and breakdown (Table 5) increased in winter (December-February) as the day length and temperature decreased in the field (Fig 6). This expression pattern agreed with that reported previously for the cambial region of Japanese cedar [26]. When cambial activity and new xylem formation are activated between March and October, most homologs of starch degradation-related genes in the cambial region exhibit minimum expression [26]. Seasonal dynamics of carbohydrate storage may be controlled by these starch-related genes. Seasonal dynamics of carbohydrate storage in Japanese cedar seedlings has been studied previously by measuring starch and sugar concentrations in the upper, middle, and lower parts of shoots and rootlets [45]. The starch concentration exhibited a small peak in autumn (November) and a large peak in spring (May), and the sugar concentration exhibited a peak in winter (January) in every part of the seedlings. The starch synthesized in autumn may be converted into sugar during the winter to enable survival in the harsh environment. These phenomena are in agreement with the annual expression pattern of starch-related genes observed in this study.

The annual dynamics of starch-related gene expression may be affected by environmental factors. In *Populus* and *Picea glauca* (white spruce), starch synthesis- and breakdown-related genes are induced by shorter day length [8, 9, 46]. In *P*. *glauca*, starch-synthesis genes such as starch synthase, ADP-glucose pyrophosphorylase large subunit, and starch branching enzyme are up-regulated under short day conditions [46]. However, only the *starch synthase 3* gene was up-regulated in Japanese cedar under SD conditions in the present study (Table 5). Previous reports indicated that the level of freezing tolerance induced by short day- or low temperature-conditions varies with plant species [47–50]. As the expression of starch-related genes may be closely related to freezing tolerance, variations between species in the expression levels of starch-related genes under SD conditions may indicate that different processes are involved in induction of freezing tolerance. Only 2 of 18 targets related to starch synthesis and 4 of 24 targets related to starch breakdown were up-regulated under SD conditions (14.3%). Although the temperature under the LT condition was not as low as the winter temperature, more genes were up-regulated under these conditions (33.3%). Low temperature may play an important role in regulating the expression of starch-related genes and the induction of freezing tolerance in Japanese cedar.

### Comparing the expression pattern of core clock-related genes to other species

**Fig 7:**
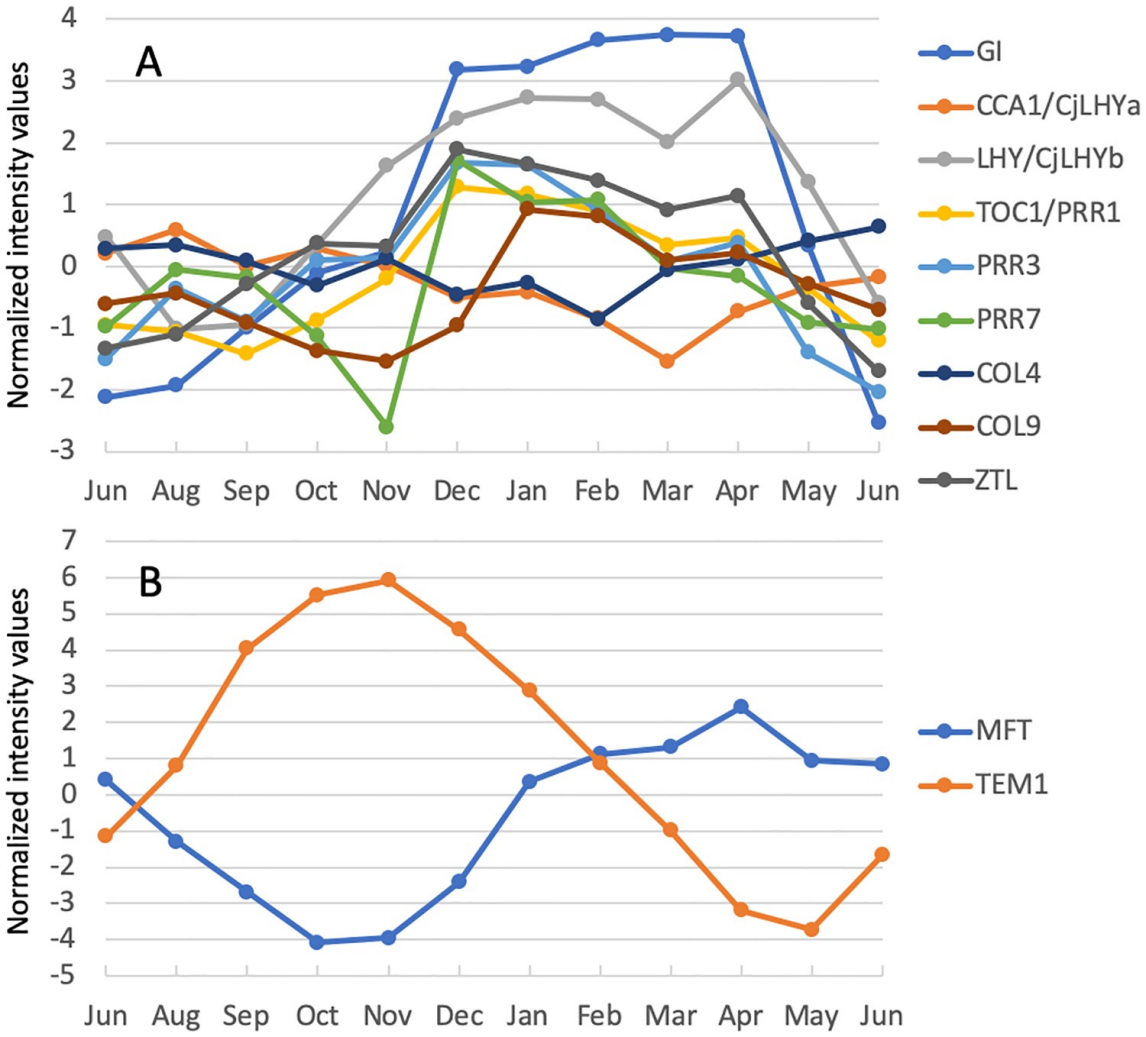
Annual expression pattern of clock-related genes and PEBP family-related genes under field conditions.

Photoperiod sensing by light receptors is closely related to the circadian clock [5, 51]; the diurnal pattern of the transcriptome changes in response to photoperiod variations, and the amplitude of the cycles of clock components is influenced by temperature [52–54]. Among the nine clock-related genes analyzed in this study, seven were affected by SD conditions (Table 6). *LHY/CjLHYb* (late elongated hypocotyl, *CjLHYb* in [27]) and *ZTL* (zeitlupe) were up-regulated, and *PRR3* (pseudo-response regulator 3) and *COL9* (constans-like 9) were down-regulated under LT conditions. Most clock-related genes exhibited high expression in December-January, except *CCA1/CjLHYa* (circadian clock associated 1, *CjLHYa* in [27]) and *COL4* (constans-like 4) (Fig 7). The high expression of these genes in winter was consistent with our previous report that found dampening of diurnal rhythms and high expression of clock genes in winter [27]. The *GI* (gigantea), *LHY/CjLHYb*, and *ZTL* genes exhibited correlation coefficients >0.8 and were located in close proximity in the co-expression gene network, suggesting that they play similar roles in modulating annual dynamics (Fig 5). In contrast, *PRR3* and *PRR7* (pseudo-response regulator 7) were located at a distance from these genes.

**Table 6:** Effects of day length and temperature on clock- and PEBP family-related genes.

sequence ID	*Arabidopsis* ID	e-value	gene	description	day length		temperature	
					up/down*	cluster	up/down*	cluster
**clock-related genes**								
reCj10286ex_ne:-L-W:isotig10253	AT1G22770	1.36E-113	*GI*	gigantea protein	-	-	-	-
reCj02587ex_ne:-LSW:isotig02565	AT2G46830	1.33E-33	*CCA1/CjLHYa*	circadian clock associated 1	down	2	-	-
reCj08322ex_ne:-LSW:isotig08289	AT1G01060	1.94E-32	*LHY/CjLHYb*	Homeodomain-like superfamily protein	down	2	up	4
reCj03391ex_ne:---W:isotig03366	AT5G61380	2.09E-93	*TOC1/PRR1*	CCT motif -containing response regulator protein	down	2	-	-
reCj09000ex_ne:----:isotig08967	AT5G60100	5.60E-56	*PRR3*	pseudo-response regulator 3	up	3	down	1
reCj03546ex_ne:MLSW:isotig03520	AT5G02810	7.40E-88	*PRR7*	pseudo-response regulator 7	up	3	-	-
reCj15175ex_ne:MLS-:isotig15142	AT5G24930	1.25E-95	*COL4*	CONSTANS-like 4	-	-	-	-
reCj21135ex_ne:----:isotig21102	AT3G07650	2.14E-23	*COL9*	CONSTANS-like 9	down	2	down	2
reCj08117ex_ne:MLSW:isotig08084	AT5G57360	0	*ZTL*	Galactose oxidase/kelch repeat superfamily protein	up	3	up	4
**PEBP family-related genes**								
reCj16177ex_ne:-LSW:isotig16144	AT1G18100	5.10E-84	*MFT*	PEBP (phosphatidylethanolamine-binding protein) family protein	down	2	down	2
reCj12017ex_ne:----:isotig11984	AT1G25560	2.86E-82	*TEM1*	AP2/B3 transcription factor family protein	up	3	up	4

*Indicates up- or down-regulation under SD (day length) or LT (temperature) conditions.

Interestingly, the expression patterns of the core clock-related genes differed in comparison to other species reported previously. Whereas *LHY* is reportedly up-regulated under SD conditions in *Populus* [8], both homologs of *LHY* (*LHY/CjLHYb* and *CCA1*/*CjLHYa*) were significantly down-regulated under SD conditions in Japanese cedar (Table 6). Moreover, although no annual expression rhythms were reported for *LHY* and *TOC1* (timing of Cab expression 1) in *P*. *menziesii* [19], one of the *LHY* homologs (*LHY/CjLHYb*) and *TOC1* exhibited annual rhythms and high expression in winter in Japanese cedar (Fig 7). Differences in the diurnal expression pattern of core clock-related genes between *P*. *menziesii*, *Arabidopsis*, and Japanese cedar were described in a previous report [19]. These differences in diurnal rhythms may reflect differences in annual expression patterns. These results suggest that different regulatory mechanisms exist between species, which may influence the annual growth pattern.

The expression of two *COL* genes identified in the conifer *P*. *abies* (*PaCOL1* and *PaCOL2*) was shown to decrease significantly in needles and shoot tips under SD conditions prior to growth cessation and bud formation [55]. It was hypothesized that *PaCOL1* and *PaCOL2* are not functional homologues of *Arabidopsis CO* [5], which promotes flowering in response to long day conditions [56]. The classification of *PaCOL1* and *PaCOL2* and their expression pattern were similar to *COL* of the moss *Physycomitrella* (*PpCOL1*) [5, 57]. Our results agree with the reported expression pattern of *COL* genes in *P*. *abies* [55], indicating the conservation of regulatory mechanisms among conifer species. Expression of the putative *COL9* gene in Japanese cedar was down-regulated under SD conditions (Table 6), decreased from August to November, and increased sharply in January under field conditions (Fig 7). In contrast, no differences in *COL4* expression were observed between SD/LD and LT/HT conditions, with stable expression throughout the year (Table 6, Fig 7).

### *MFT* may play an important role in day length and temperature responses in Japanese cedar

The *FT* gene, which belongs to the PEBP gene family, is a key integrator of day length and temperature signals in trees [11–14]. Interestingly, *MFT*, which is also a member of the PEBP gene family, may play a role in Japanese cedar similar to *FT*. *MFT* expression was down-regulated under both SD and LT conditions (Table 6), decreasing from June to November (Fig 7B), indicating the possibility that *MFT* regulates growth rhythms in Japanese cedar, similar to *PtFT1* in *Populus*. *PtFT1* is down-regulated in the early stage of the SD response and regulates short day-induced growth cessation and bud setting in *Populus* [11]. The gene co-expression network indicated an inverse correlation between *MFT* and *TEM1*, which encodes a homolog of a direct repressor of *FT* in *Arabidopsis* [58] (Figs 5 and 7B). As in Japanese cedar, down-regulation of *MFT* under SD conditions (Table 6) was observed in the pteridophyte *Adiantum capillus-veneris* (Ac*MFT*) [59]. Also, high *MFT* expression in leaves was observed during the vegetative stage, similar to Japanese cedar (Fig 7B). Ectopic expression of Ac*MFT* in *Arabidopsis* suggests that the gene functions similarly to *FT* in flowering plants [59]. Based on phylogenetic analyses of plant MFT amino acid sequences, *MFT* of Japanese cedar is similar to *PaMFT1* and *PaMFT2* of *P*. *abies*, in which their expression is not correlated with bud setting [12, 14] (S5A Fig). In *P*. *abies*, *PaFTL2*/*PaFT4* expression increases after plants are transferred to SD conditions, in contrast to *Populus*, and the gene regulates bud setting and annual growth rhythm [12, 14]. In wheat and *Arabidopsis*, MFT promotes seed dormancy [60, 61]. A previous study reported that the expression of *MFT* did not change under SD conditions during a 6-week experiment in *Populus* [8], whereas another study reported that expression increased for 28 days and subsequently decreased under SD conditions in *P*. *glauca* [44]. In contrast to *PtFT1*, *PaFTL2/PaFT4*, and Ac*MFT*, *MFT* in Japanese cedar did not exhibit a diurnal rhythm in July in our study (S5B Fig). These results are suggestive of evolutionary differentiation in the function of PEBP gene family members. Although we collected sequence data from various parts of trees in various developmental stages and seasons (19 libraries, approximately 3 million reads, 34,731 isotigs in total) using an NGS approach [28], no homolog of *FT* in Japanese cedar was identified.

## Conclusion

This study provides considerable insight into the control of phenology in Japanese cedar, a gymnosperm indeterminate species. Our microarray data demonstrated dynamic annual changes in the transcriptome and the significant contribution of SD- and LT-regulated genes. More than half of the top 1,000 up-regulated targets in the growth/dormant period in the field were regulated by day length or temperature solely, indicating that interaction between day length and temperature is not necessary for their expression. Interestingly, we found that SD and LT conditions generally regulate different genes. However, the co-expression network of annual transcriptome dynamics revealed a close relationship between SD- and LT-regulated genes. These results indicate that the respective effects of SD and LT conditions play different roles but interact to regulate annual transcriptome dynamics. Compared with previous reports in other plant species, Japanese cedar exhibited several unique characteristics. Although starch-related genes are known to be regulated by day length in other species, only a few starch-related genes were up-regulated in Japanese cedar. Upstream signaling pathway genes in the clock and PEBP family also exhibited unique expression patterns. Our data indicate that there are different seasonal regulatory mechanisms among tree species. There are several possible reasons for the variation among species: traits obtained during evolution (i.e., gymnosperms and angiosperms), differences in seasonal growth pattern (i.e., determinate and indeterminate species), and adaptation to environmental conditions in the distribution area. As our results indicate that not all regulatory mechanisms are conserved among species, investigations in other tree species would be of interest.

## Supporting information

S1 Fig Annual changes in temperature and day length under natural conditions. (A) Temperature data at Hitachi (36°34’N 140°38’E 34 m, approximately 15 km from the sampling site) were provided by the Japan Meteorological Agency (http://www.jma.go.jp/jma/index.html). Each line represents 1 week’s average maximum, average, and minimum temperatures. (B) Day length was calculated by the time of sunrise and sunset provided by the National Astronomical Observatory of Japan (http://eco.mtk.nao.ac.jp/cgi-bin/koyomi/koyomix.cgi).
(TIFF)

S2 Fig Annual height growth of Japanese cedar under natural conditions. (TIFF)

S3 Fig Expression patterns of five selected targets analyzed by microarrays and qRT-PCR. Blue line represents the average normalized intensity value as determined by microarray analysis, and red line represents the average relative normalized transcript abundance as determined by qRT-PCR. Dots represent the normalized intensity value and relative normalized transcript abundance of each sample.
(TIFF)

S4 Fig Growth in height and projected area of cuttings grown in chambers with controlled day length and temperature. Growth rate was calculated by dividing growth by the value at the start of the experiment. Asterisks indicate statistically significant differences between the two conditions (*P<0.05, **<0.01, ***<0.001).
(TIFF)

S5 Fig Phylogenetic analysis of *MFT* genes in plants (A) and diurnal expression of *MFT* in Japanese cedar. (A) The neighbor-joining method [32] was used to construct the phylogenetic trees. The species names are abbreviated as follows: At, *Arabidopsis thaliana* (thale cress); Pt, *Populus trichocarpa* (black cottonwood); Os, *Oryza sativa* (Japanese rice); Pa, *Picea abies* (Norway spruce); Ps, *Picea sitchensis* (Sitka spruce); Cj, Japanese cedar (*Cryptomeria japonica*); Ac, *Adiantum capillus-veneris* (pteridophyte); Pp, *Physcomitrella patens* subsp. patens (moss). The number following the species name indicates its NCBI accession number. (B) Diurnal expression of *MFT* in summer (July 30–31, 2012), analyzed using qRT-PCR as described in [27].
(TIFF)

S1 Table Primers used for quantitative RT-PCR in this study. *Reference [27].
(XLSX)

S2 Table Top-scoring targets for principal component 1. SD-up, SD-down, LT-up, and LT-down indicate SD up-regulated targets, SD down-regulated targets, LT up-regulated targets, and LT down-regulated targets, respectively.
(XLSX)

S3 Table Day length- and temperature-regulated targets. (XLSX)
