# Supplementary figures and images for: Effects of day length- and temperature-regulated genes on annual transcriptome dynamics in Japanese cedar (Cryptomeria japonica D. Don), a gymnosperm indeterminate species
Source: PLoS One. 2020 Mar 9;15(3):e0229843. doi: 10.1371/journal.pone.0229843 (PMC7062269; doi:10.1371/journal.pone.0229843)

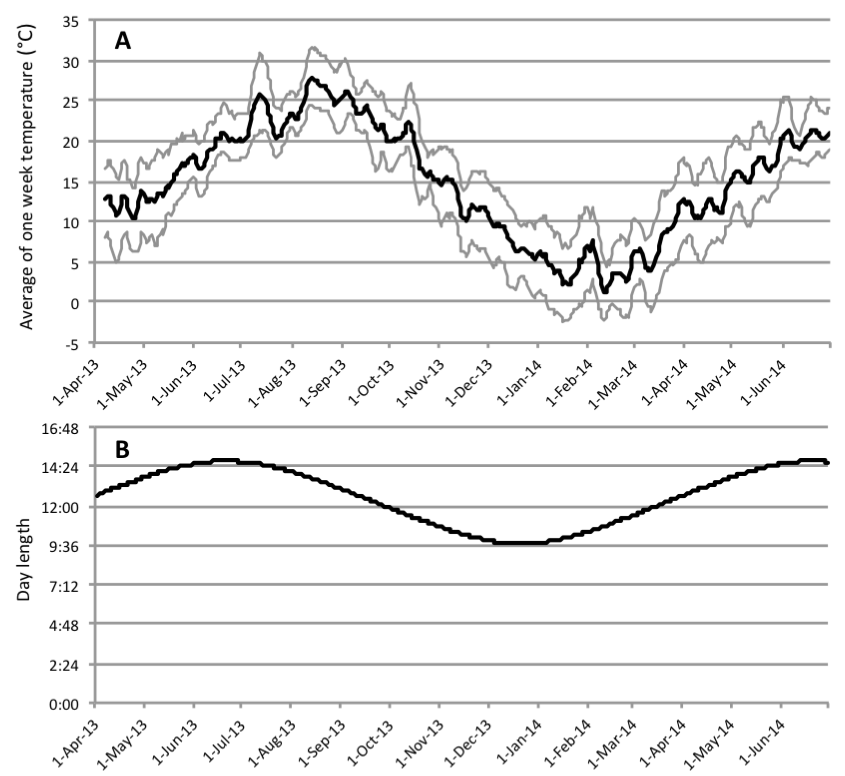

Supplement: S1 Fig — (A) Temperature data at Hitachi (36°34’N 140°38’E 34 m, approximately 15 km from the sampling site) were provided by the Japan Meteorological Agency (http://www.jma.go.jp/jma/index.html). Each line represents 1 week’s average maximum, average, and minimum temperatures. (B) Day length was calculated by the time of sunrise and sunset provided by the National Astronomical Observatory of Japan (http://eco.mtk.nao.ac.jp/cgi-bin/koyomi/koyomix.cgi). (TIFF) [file pone.0229843.s001.tiff]

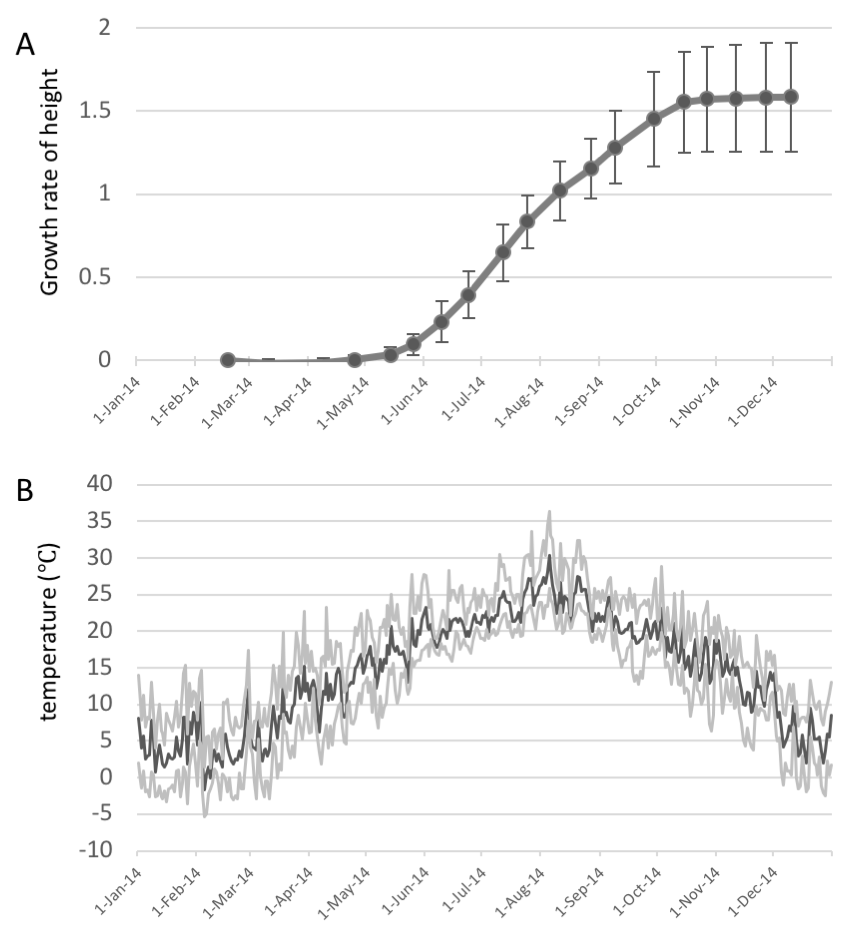

Supplement: S2 Fig — (TIFF) [file pone.0229843.s002.tiff]

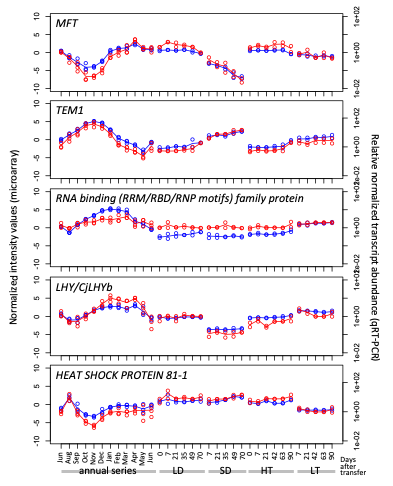

Supplement: S3 Fig — Blue line represents the average normalized intensity value as determined by microarray analysis, and red line represents the average relative normalized transcript abundance as determined by qRT-PCR. Dots represent the normalized intensity value and relative normalized transcript abundance of each sample. (TIFF) [file pone.0229843.s003.tiff]

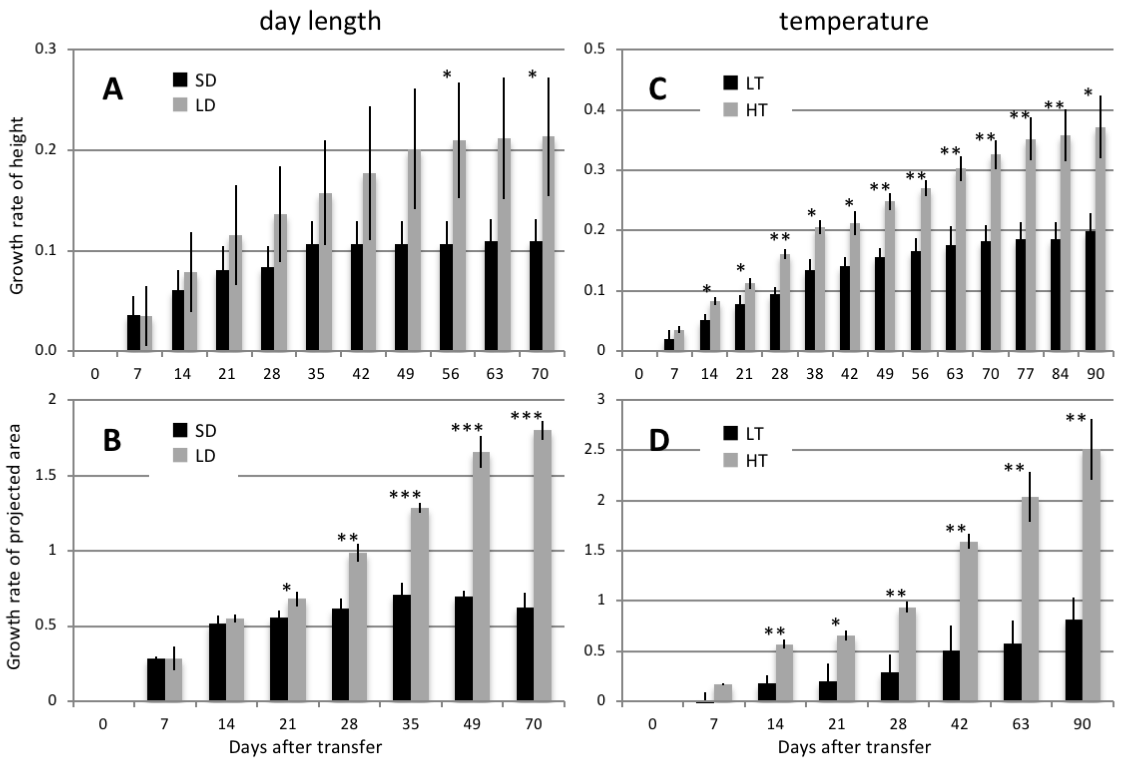

Supplement: S4 Fig — Growth rate was calculated by dividing growth by the value at the start of the experiment. Asterisks indicate statistically significant differences between the two conditions (*P<0.05, **<0.01, ***<0.001). (TIFF) [file pone.0229843.s004.tiff]

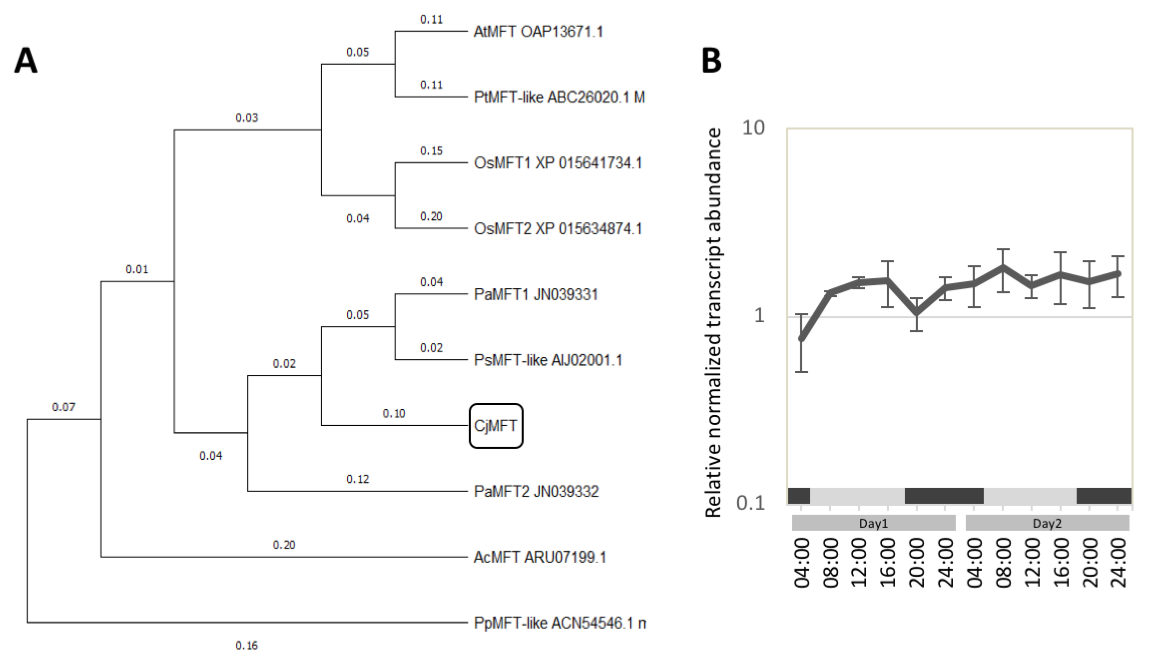

Supplement: S5 Fig — (A) The neighbor-joining method [32] was used to construct the phylogenetic trees. The species names are abbreviated as follows: At, Arabidopsis thaliana (thale cress); Pt, Populus trichocarpa (black cottonwood); Os, Oryza sativa (Japanese rice); Pa, Picea abies (Norway spruce); Ps, Picea sitchensis (Sitka spruce); Cj, Japanese cedar (Cryptomeria japonica); Ac, Adiantum capillus-veneris (pteridophyte); Pp, Physcomitrella patens subsp. patens (moss). The number following the species name indicates its NCBI accession number. (B) Diurnal expression of MFT in summer (July 30–31, 2012), analyzed using qRT-PCR as described in [27]. (TIFF) [file pone.0229843.s005.tiff]
